# New stopping criteria for iterative root finding

**DOI:** 10.1098/rsos.140206

**Published:** 2014-10-15

**Authors:** Jorgen L. Nikolajsen

**Affiliations:** Faculty of Computing, Engineering and Sciences, Staffordshire University, Stafford ST18 0AD, UK

**Keywords:** stopping criteria, root finding, fractional significant digits

## Abstract

A set of simple stopping criteria is presented, which improve the efficiency of iterative root finding by terminating the iterations immediately when no further improvement of the roots is possible. The criteria use only the function evaluations already needed by the root finding procedure to which they are applied. The improved efficiency is achieved by formulating the stopping criteria in terms of fractional significant digits. Test results show that the new stopping criteria reduce the iteration work load by about one-third compared with the most efficient stopping criteria currently available. This is achieved without compromising the accuracy of the extracted roots.

## 2. Introduction

Stopping criteria for root finding procedures for nonlinear functions fall into two categories: (1) those that rely on the user to specify a tolerance within which the roots are needed and (2) those that seek to terminate the iterations automatically when an iterate has been reached whose accuracy cannot be improved. Both categories are widely used (e.g. [1]). Category (1) is easy to implement using stopping criteria such as |*x*_*i*_−*x*_*i*−1_|<*e* or |*x*_*i*_−*x*_*i*−1_|/|*x*_*i*−1_|<*e*, where *x*_*i*_ and *x*_*i*−1_ are successive iterates and *e* is a user-supplied upper limit on the absolute or relative error. The drawback of such stopping criteria is that they shift the responsibility for providing accurate results from the program developer to the user. Also, functions exist for which such stopping criteria fail (see Donovan *et al.* [2]). Category (2) stopping criteria avoid these limitations, but present a greater challenge to the program developer. They are the subject of this paper.

Several category (2) stopping criteria already exist. They are reviewed in §3. The new stopping criteria are then presented in §§4, 5 and 6. Finally, the new and the old criteria are compared in §7 in terms of efficiency and accuracy.

## 3. Existing stopping criteria

### 3.1 Igarashi's stopping criterion for polynomials

Igarashi [3] provides the following category (2) stopping criterion for finding the roots of the polynomial

$$p(z)=a_{n}z^{n}+a_{n-1}z^{n-1}+\cdots +a_{2}z^{2}+a_{1}z+a_{0}:$$

iterate *z*_*i*_ is declared a root if

$$|A(z_{i})-B(z_{i})|\geq \text{min}(|A(z_{i})|,|B(z_{i})|).$$

*A*(*z*_*i*_) and *B*(*z*_*i*_) are both equal to *p*(*z*_*i*_) but with *p*(*z*_*i*_) calculated in different ways: *A*(*z*_*i*_)=*p*(*z*_*i*_) with *p*(*z*_*i*_) evaluated as usual by Horner's method. *B*(*z*_*i*_) must be written as *B*(*z*_*i*_)=*D*(*z*_*i*_)−*C*(*z*_*i*_), where *D*(*z*)=*zp*^′^(*z*) and *C*(*z*)=*zp*^′^(*z*)−*p*(*z*). Both polynomials *D*(*z*) and *C*(*z*) must be reduced analytically to their simplest form and evaluated separately by Horner's method before *B*(*z*_*i*_)=*D*(*z*_*i*_)−*C*(*z*_*i*_) is calculated. Analytical reduction results in

$$D(z)=na_{n}z^{n}+(n-1)a_{n-1}z^{n-1}+\cdots +2a_{2}z^{2}+a_{1}z=0$$

and

$$C(z)=(n-1)a_{n}z^{n}+(n-2)a_{n-1}z^{n-1}+\cdots +a_{2}z^{2}-a_{0}.$$

Igarashi gives only an abbreviated explanation of how this works. Presumably, as *A*(*z*_*i*_) and *B*(*z*_*i*_) approach zero, |*A*(*z*_*i*_)−*B*(*z*_*i*_)| will initially be smaller than either |*A*(*z*_*i*_)| or |*B*(*z*_*i*_)|, thus preventing the stopping criterion from being satisfied. But, as |*A*(*z*_*i*_)| and |*B*(*z*_*i*_)| grow smaller, round-off errors will dominate |*A*(*z*_*i*_)−*B*(*z*_*i*_)| before they dominate |*A*(*z*_*i*_)| and |*B*(*z*_*i*_)|, thus providing an opportunity for the stopping criterion to be satisfied when |*A*(*z*_*i*_)−*B*(*z*_*i*_)| has lost all its significant digits.

If a third order iteration procedure, such as Laguerre's method (e.g. Orchard [4]), is used, five function evaluations are needed per iteration: *p*(*z*_*i*_), *p*^′^ (*z*_*i*_) and *p*^′′^(*z*_*i*_) are needed by the iteration procedure itself, and *D*(*z*_*i*_) and *C*(*z*_*i*_) are needed by the stopping criterion. Thus, Igarashi's stopping criterion adds two-thirds to the work load per iteration. The criterion is included in the comparisons reported in §7.

### 3.2 Igarashi's stopping criterion for nonlinear functions

Igarashi [5] provides a similar category (2) stopping criterion for general nonlinear functions, *f*(*z*): iterate *z*_*i*_ is declared a root if

$$|A(z_{i})-B(z_{i})|\geq W\;\text{min}(|A(z_{i})|,|B(z_{i})|).$$

*A*(*z*_*i*_) and *B*(*z*_*i*_) are both equal to *f*(*z*_*i*_) but with *f*(*z*_*i*_) calculated in different ways: *A*(*z*_*i*_)=*f*(*z*_*i*_) with *f*(*z*_*i*_) in its standard analytical formulation. *B*(*z*_*i*_) must be written as *B*(*z*_*i*_)=*D*(*z*_*i*_)−*C*(*z*_*i*_), where *D*(*z*)=*zf* ′(*z*) and *C*(*z*)=*zf* ′(*z*)−*f*(*z*). Both functions *D*(*z*) and *C*(*z*) must be reduced analytically to their simplest form before *B*(*z*_*i*_)=*D*(*z*_*i*_)−*C*(*z*_*i*_) is calculated. *W* must be equal to either 1.0 or 0.5 if *f*(*z*) is algebraic, and *W* must be equal to 0.01 when *f*(*z*) is transcendental. It is not clear what value *W* should have if *f*(*z*) has both algebraic and transcendental terms. Igarashi gives only an abbreviated explanation of how this works. Presumably, the basic explanation is similar to the one proposed above for polynomials. Igarashi also gives only an abbreviated explanation for the choice of *W*. *W*=1.0 appears to be based on the assumption that the evaluations of algebraic functions and polynomials incur similar round-off errors, so the same stopping criterion can be used for both. But Igarashi also suggests that *W*=0.5 can be used instead to relax the stopping criterion to allow for errors in experimental data and/or the conversion of experimental data from decimal to floating-point binary. Finally, Igarashi chooses *W*=0.01 to further relax the stopping criterion for transcendental functions in order to account for the truncation errors incurred by the intrinsic transcendental functions used to evaluate *A*(*z*_*i*_), *D*(*z*_*i*_) and *C*(*z*_*i*_).

If a third order iteration procedure, such as Ostrowski's method (e.g. Orchard [4]), is used, five function evaluations are needed per iteration: *f*(*z*_*i*_), *f*^′^ (*z*_*i*_) and *f*^′′^(*z*_*i*_) are needed by the iteration procedure itself, and *D*(*z*_*i*_) and *C*(*z*_*i*_) are needed by the stopping criterion. Thus, Igarashi's stopping criterion adds two-thirds to the work load per iteration. The criterion is included in the comparisons reported in §7.

### 3.3 Adams' and Grant & Hitchins' stopping criteria for polynomials

A category (2) stopping criterion for polynomial root finding was proposed by Adams [6]. It was extended by Grant & Hitchins [7] to include polynomials with complex coefficients. Using the extended criterion, the iterate *z*_*i*_=*x*_*i*_+*jy*_*i*_ is accepted as a root of the complex polynomial

$$p(z)=(a_{n}+jb_{n})z^{n}+(a_{n-1}+jb_{n-1})z^{n-1}+\cdots +(a_{1}+jb_{1})z+a_{0}+jb_{0}$$

if |*c*_0_|<*ϵg*_0_(1+*ϵ*)^5*n*^ and |*d*_0_|<*ϵh*_0_(1+*ϵ*)^5*n*^. *c*_0_, *d*_0_, *g*_0_ and *h*_0_ derive from the recurrences:

$$\left\{\begin{array}{c} c_{n}=a_{n}\\ d_{n}=b_{n}\\ g_{n}=1\\ h_{n}=1 \end{array}\right\}\left\{\begin{array}{c} c_{k}=x_{i}c_{k+1}-y_{i}d_{k+1}+a_{k}\\ d_{k}=y_{i}c_{k+1}-x_{i}d_{k+1}+b_{k}\\ g_{k}=|x_{i}|(g_{k+1}+|c_{k+1}|)+|y_{i}|(h_{k+1}+|d_{k+1}|)+|a_{k}|+2|c_{k}|\\ h_{k}=|y_{i}|(g_{k+1}+|c_{k+1}|)+|x_{i}|(h_{k+1}+|d_{k+1}|)+|b_{k}|+2|d_{k}| \end{array}\right\},k=n-1,n-2,\ldots ,0.$$

*ϵ*=2^−*t*^ is the machine epsilon, i.e. *t* is the bit length of the floating-point significand, e.g. *t*=53 for IEEE double-precision. A rigorous derivation of this stopping criterion is given by Grant & Hitchins [7]. *c*_0_+*jd*_0_ is *p*(*z*) calculated by Horner's method. *ϵg*_0_(1+*ϵ*)^5*n*^ and *ϵh*_0_(1+*ϵ*)^5*n*^ are error bounds on *c*_0_ and *d*_0_, respectively, with allowances made for any additional rounding errors caused by the calculation of *g*_0_ and *h*_0_.

*c*_0_ and *d*_0_ are needed by both the iteration procedure itself and by the stopping criterion, whereas *g*_0_ and *h*_0_ are needed by the stopping criterion only. Inspection of the above recurrences shows that the calculation of *g*_0_ and *h*_0_ requires more than twice the work load of the calculation of *c*_0_ and *d*_0_. Thus, for a third order iteration procedure (requiring both *p*(*z*), *p*^′^(*z*) and *p*^′′^(*z*)), Grant & Hitchins' stopping criterion adds more than two-thirds to the work load. The criterion is included in the comparisons reported in §7.

### 3.4 Garwick's and Ward's stopping criteria for nonlinear functions

Garwick [8] proposed the following very simple category (2) stopping criterion: *z*_*i*_ is a root of the nonlinear function *f*(*z*) if *e*_*i*_>*e*_*i*−1_ or *e*_*i*−1_=0, where *e*_*i*_=|*z*_*i*_−*z*_*i*−1_| and *e*_*i*−1_=|*z*_*i*−1_−*z*_*i*−2_|. *z*_*i*−2_, *z*_*i*−1_ and *z*_*i*_ are successive iterates. The criterion loosely states that a root has been found when the iteration increment *e*_*i*_ starts to increase. The precondition *e*_*i*_<*e*_0_ is required to ensure that convergence has started before the stopping criterion is applied. *e*_0_ is a user-supplied ‘small’ number. Garwick's criterion is based on the assumption that once convergence has started, the rate of convergence does not decrease until a root has been found.

As it stands, Garwick's stopping criterion is inadequate because the precondition *e*_*i*_<*e*_0_ places a limit on the absolute error *e*_*i*_ only, which is insufficient when roots with both large and small absolute values are present. Ward [9] suggested the following stopping criterion, which helps to overcome that problem:

$$\begin{array}{rl} & z_{i-1}\text{ is a root if }e_{i}>e_{i-1}.\text{ Preconditions: }(1)\;e_{i}\leq 10^{-7}\text{ if }|z_{i-1}|<10^{-4}\\ & \;\text{and }(2)\;e_{i}/|z_{i-1}|\leq 10^{-3}\text{ if }|z_{i-1}|\geq 10^{-4}. \end{array}$$

Precondition (1) states that when |*z*_*i*−1_| is less than 10^−4^, convergence is deemed to have started when the *absolute* distance between successive iterates is less than or equal to 10^−7^. Precondition (2) states that when |*z*_*i*−1_| is greater than or equal to 10^−4^, convergence is deemed to have started when the *relative* distance between successive iterates is less than or equal to 10^−3^.

Note that Ward's stopping criterion requires only the function evaluations already needed by the iteration procedure itself. On the other hand, Ward always requires at least one post-convergence iteration to confirm that the best possible root has been found. Ward's criterion is included in the comparisons reported in §7.

### 3.5 Other stopping criteria

Vignes [10] developed a statistically based category (2) stopping criterion, which is part of the CADNA software library (e.g. [11]). Vignes' criterion has the advantage of providing accurate estimates of the round-off errors incurred, but it also has the following drawbacks: (i) CADNA runs only on LINUX and UNIX based operating systems, (ii) complex arithmetic is not supported, and (iii) ‘code which uses CADNA runs at least three times slower than [without]’ (quote Jézéquel *et al.* [11]). These limitations are deemed to make Vignes' stopping criterion too restrictive and too slow to be included in the comparisons in §7.

Brent [12] proposed a simple category (2) stopping criterion specifically for his widely used procedure for finding the real roots of relatively badly behaved real, nonlinear functions. The robustness of Brent's procedure is achieved at the expense of convergence rate, which is typically less than quadratic. By contrast, the new stopping criteria, outlined in this paper, are most useful when applied to iteration procedures with high rates of convergence (at least quadratic but preferably cubic or higher). Brent's procedure is therefore deemed to be too slow to be included in the comparisons in §7.

## 4. The new stopping criteria

The new stopping criteria aim to maximize the efficiency of high order root finding procedures without compromising the accuracy of the roots. This is achieved (i) by eliminating the need for function evaluations that are not required by the iteration procedure itself and (ii) by immediately terminating the iterations when no further improvement of the roots is possible. The idea behind the new stopping criteria is outlined in Nikolajsen [13], but a satisfactory implementation has only recently been made possible by the development of a procedure for high-accuracy calculation of fractional significant digits [14].

A total of four new stopping criteria are needed, as outlined in §4.1 through 4.4. The first three provide the efficiency improvements. The fourth catches roots that are too ill-conditioned, or whose convergence rate is too slow, to invoke the first three. The four criteria are derived in §4 with the assumption that they are being used to find non-zero, real roots *z* of real functions *f*(*z*). Section 5 shows how the criteria can be readily adapted to finding zero roots. Section 6 shows how they can be applied in the complex domain.

As an introduction to the derivation of the new stopping criteria, the operation of Garwick's criterion is illustrated by the iteration sequence shown in table 1. Inspection of table 1 shows that the distance *e*_*i*_ between the iterates decreases steadily, indicating that convergence is underway. *e*_*i*_ reaches a minimum at *e*_4_=0.000002, then grows to *e*_5_=0.000003, indicating that both *z*_4_ and *z*_5_ lie in the area of indeterminacy (AOI) of the root, so either can be declared a root. (Garwick's criterion makes *z*_5_ the root but experience shows that *z*_4_ is often the more accurate.) But if *z*_4_ and *z*_5_ are equally valid as a root, *z*_3_ must also be equally valid because it lies between *z*_4_ and *z*_5_. In other words, *z*_3_ can be declared the root, and *z*_4_ and *z*_5_ are redundant except as confirmation that *z*_3_ is a root. Thus, Garwick's need for post-convergence iterates *z*_4_ and *z*_5_ has increased the work load by two-thirds. The new stopping criteria will eliminate this redundancy, as explained in §4.1.

**Table 1.** Application of Garwick's stopping criterion.

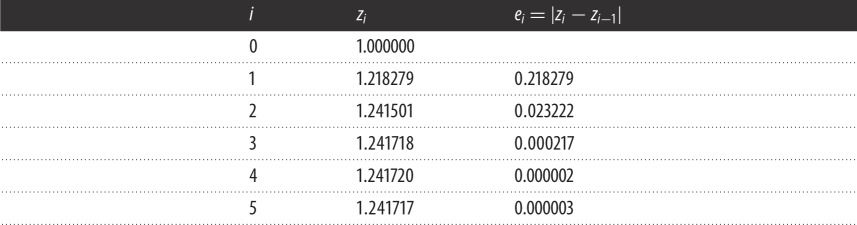

Note also that Garwick's stopping criterion puts no limit on the number of post-convergence iterations needed. One possible scenario is *z*_5_=1.241719, leading to *e*_5_=0.000001, which is less than *e*_4_, so at least one additional iterate, *z*_6_, would be needed. A *z*_6_-value of 1.241718 would then confirm that *z*_3_=1.241718 is a root. In other words, three iterations are needed to reach the root but Garwick requires three additional iterations to confirm that the root has been reached.

### 4.1 Stopping criterion #1

$$z_{i}\text{ is a root if }s_{i}^{2}/s_{i-1}\geq s_{m}.\text{ Precondition}:\;s_{i-1}\geq s_{m}/q_{m}^{2}.$$

Here, *z*_*i*_ is the *i*th iterate in the iteration sequence generated by the iteration procedure to which the stopping criterion is applied. *s*_*i*_ is the number of matching leading bits (MLBs) of the two successive iterates *z*_*i*−1_ and *z*_*i*_. *s*_*i*−1_ is defined similarly. *s*_*i*_ and *s*_*i*−1_ can be calculated as outlined in Nikolajsen [14]. The calculation is demonstrated below.

Also in stopping criterion #1, *s*_*m*_ is the length of the floating-point significand used, e.g. *s*_*m*_=53 bits and *s*_*m*_=113 bits, respectively, for IEEE double-precision and quad-precision. *q*_*m*_ is the order of the iteration procedure to which the stopping criterion is applied, e.g. *q*_*m*_=3 for Laguerre's method and Ostrowski's method.

Stopping criterion #1 is explained with reference to table 2*a*,*b*. These examples use iterates *z*_1_, *z*_2_ and *z*_3_ from table 1 but written in binary notation with a significand-length of *s*_*m*_=24 bits, as in IEEE single-precision. Also shown are the corresponding iteration increments *e*_2_=|*z*_2_−*z*_1_| and *e*_3_=|*z*_3_−*z*_2_|. A trailing ‘*b*’ designates a binary number.

**Table 2.** Convergence by stopping criteria #1 and #2.

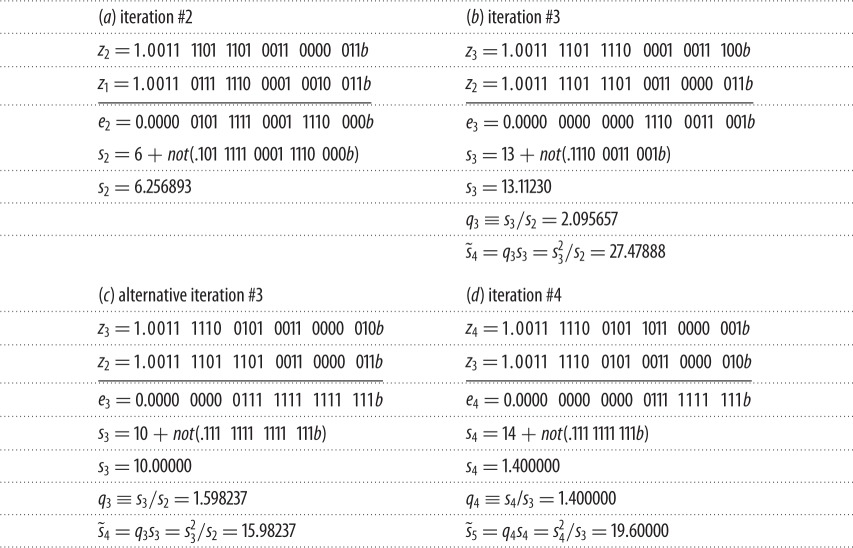

*s*_2_ in table 2*a* is the number of MLBs of *z*_1_ and *z*_2_ following iteration #2. *s*_2_ is equal to the number of leading zero bits of *e*_2_ (i.e. 6) plus a fraction produced by (i) removing the radix point and the leading zeros from *e*_2_ to produce the bit-string 101 1111 0001 1110 000b, (ii) taking two's complement of the bit-string to get 010 0000 1110 0001 111*b*, and (iii) placing a radix point in front of the complement to produce the binary fraction 0.010 0000 1110 0001 111*b* = 0.256893. This fraction is added to the number of leading zeros of *e*_2_ to get *s*_2_=6+0.256893=6.256893, as shown in table 2*a*. *s*_2_=6.256893 states that *z*_1_ and *z*_2_ have 6.256893 leading bits in common. The *not*() function in table 2*a* is used as shorthand for the above procedure for finding the fractional part of *s*_2_. A simple numerical procedure for calculating *s* is outlined in Nikolajsen [14]. The code is available from the author on request.

Moving now to table 2*b*, the number of MLBs can be seen to have increased to *s*_3_=13.11230 following completion of iteration #3. With *s*_2_=6.256893 and *s*_3_=13.11230, the rate at which MLBs are being gained is *q*_3_=*s*_3_/*s*_2_=2.095657. This is by definition the effective convergence rate following completion of iteration #3. *q*_3_=2.095657 shows that the convergence rate is slightly higher than quadratic. Experience shows that when the rate of convergence is rapid enough to invoke stopping criterion #1, it is also rapid enough so that it does not diminish until the AOI of the root has been reached. Thus, with the number of MLBs already increasing by a factor *q*_3_=2.095657 per iteration, the number of MLBs after the next iteration (#4) cannot be less than ${\tilde{s}}_{4}=q_{3}s_{3}=s_{3}^{2}/s_{2}=27.47888$. But that exceeds the length of the floating-point significand used (*s*_*m*_=24), so in practice, *z*_3_ and *z*_4_ will either have *s*_*m*_=24 leading bits in common or, more likely, they will both be located within the AOI of the root. In either case, no further improvement is possible, so iteration #4 can be omitted and *z*_3_ can be declared a root. Thus, only the two consecutive converging iterations, shown in table 2*a*,*b*, are needed before stopping criterion #1 is invoked.

All the *z*-values of table 2*a*,*b* are taken from table 1, so stopping criterion #1 has identified iterate *z*_3_ from table 1 as the root without requiring any of the post-convergence iterations needed by Garwick's method.

The precondition, $s_{i-1}\geq s_{m}/q_{m}^{2},$ is empirical and based on the almost self-evident assumption that the convergence rate *q*_*i*_≡*s*_*i*_/*s*_*i*−1_ is unlikely to exceed the order *q*_*m*_ of the iteration procedure used. The resulting inequality, *s*_*i*_/*s*_*i*−1_≤*q*_*m*_, together with the stopping criterion itself, $s_{i}^{2}/s_{i-1}\geq s_{m}$, lead to the precondition $s_{i-1}\geq s_{m}/q_{m}^{2}$.

Assuming that the stopping criterion is being applied to a third order root finder implemented in IEEE double-precision, then precondition $s_{i}^{2}/s_{i-1}\geq s_{m}$ and stopping criterion $s_{i-1}\geq s_{m}/q_{m}^{2}$ can be solved for *s*_*i*−1_ and *s*_*i*_ to get $s_{i-1}\geq s_{m}/q_{m}^{2}=53/3^{2}≃5.89$ MLBs≃1.8 decimal digits and *s*_*i*_≥*s*_*m*_/*q*_*m*_=53/3≃17.7 MLBs≃5.3 decimal digits. In other words, the minimum requirement for stopping criterion #1 to be invoked is that iterates *z*_*i*−2_ and *z*_*i*−1_ have at least 1.8 decimal digits in common and *z*_*i*−1_ and *z*_*i*_ have at least 5.3 decimal digits in common. These are surprisingly small numbers, considering that they imply that *z*_*i*_ is a root which cannot be improved further: either *z*_*i*_ will have *s*_*m*_=53 correct bits ≃15.9 correct decimal digits or, more likely, *z*_*i*_ will be in the AOI of the root.

### 4.2 Stopping criterion #2

$$z_{i+1}\text{ is a root if }s_{i}^{2}/s_{i-1}>s_{i+1}.\text{ Preconditions}:\;s_{i-1}\geq s_{m}/q_{m}^{2}\text{ and }s_{i}-s_{i-1}\geq s_{m}/q_{m}^{2}.$$

Stopping criterion #2 is needed when the convergence rate is not quite fast enough to trigger stopping criterion #1. The criterion is explained with reference to table 2*a*,*c*,*d*, with table 2*b* ignored. The explanation assumes familiarity with the explanation for stopping criterion #1. As shown in table 2*a*,*c*, *z*_1_ and *z*_2_ have *s*_2_=6.256893 MLBs, and *z*_2_ and *z*_3_ have *s*_3_=10.00000 MLBs. Thus, assuming that convergence has started, *z*_3_ and *z*_4_ will have at least ${\tilde{s}}_{4}=q_{3}/s_{3}=15.98237$ MLBs as shown (unless the AOI has been reached). This is less than *s*_*m*_=24, so *z*_3_ cannot be declared a root based on stopping criterion #1. Table 2*d* shows that *z*_4_ and *z*_5_ (not calculated) will have a minimum of ${\tilde{s}}_{5}=q_{4}/s_{4}=19.60000$ MLBs (unless the AOI is reached). This is also less than *s*_*m*_=24, so stopping criterion #1 cannot confirm that *z*_4_ is a root either. However, the actual number of MLBs of *z*_3_ and *z*_4_ is *s*_4_=14.00000, which is smaller than ${\tilde{s}}_{4}=15.98237$, which is the minimum number of MLBs that *z*_3_ and *z*_4_ should have in common given the convergence rate of *q*_3_=1.598237. The only possible explanation is that the AOI has been reached, thus *z*_4_ must be a root. This conclusion is based on the observation that when the convergence has become so rapid that stop criterion #2 can be invoked, it does not diminish until the AOI of the root has been reached. The AOI having been reached is also reflected by the rate of convergence diminishing from *q*_3_=1.598237 to *q*_4_=1.400000. *q*_3_>*q*_4_ is easily expanded and generalized into $s_{i}^{2}/s_{i-1}>s_{i+1}$, which is stopping criterion #2.

Both preconditions for stopping criterion #2 are empirical. Precondition $s_{i-1}\geq s_{m}/q_{m}^{2}$ is the same as for stopping criterion #1 and is based on the same considerations. The additional precondition $s_{i}-s_{i-1}\geq s_{m}/q_{m}^{2}$ is needed to compensate for the fact that the stopping criterion itself makes no contribution to ensuring that convergence is underway and sufficiently rapid to allow the stopping criterion to be applied safely. Solving the two preconditions for *s*_*i*_ results in $s_{i}\geq 2\cdot s_{m}/q_{m}^{2}$. Thus, using a third order root finder implemented in IEEE double-precision results in $s_{i-1}\geq s_{m}/q_{m}^{2}=53/3^{2}≃5.89$ MLBs ≃ 1.8 decimal digits and $s_{i}\geq 2\cdot s_{m}/q_{m}^{2}≃11.8$ MLBs ≃ 3.5 decimal digits. In other words, the minimum requirement for stopping criterion #2 to be invoked is that iterates *z*_*i*−2_ and *z*_*i*−1_ have at least 1.8 decimal digits in common and *z*_*i*−1_ and *z*_*i*_ have at least 3.5 decimal digits in common. This is even less demanding than for stopping criterion #1 but it is accompanied by the demand for an additional iteration to find *z*_*i*+1_.

### 4.3 Stopping criterion #3

Stopping criterion #3 is designed to catch roots extracted in a single iteration. This happens regularly in practice and is too fast for stopping criteria #1 and #2 to be invoked. Stopping criterion #3 must be divided into the following two sub-criteria.

#### 4.3.1 Stopping criterion #3.1

$$z_{1}\text{ is a root if }(1)\;s_{1}\geq s_{m}/2\text{ and }z_{0}\neq 0\text{ or }(2)\;s_{1}\geq s_{m}\text{ and }z_{0}=0.$$

Stopping criterion #3.1 states that the first iterate *z*_1_ can be declared a root if it has as least *s*_*m*_/2 leading bits in common with the start value *z*_0_, provided that *z*_0_ is non-zero. If *z*_0_ is zero, the stricter condition *s*_1_≥*s*_*m*_ applies.

Condition *s*_1_≥*s*_*m*_/2 ensures that *z*_0_ and *z*_1_ have at least *s*_*m*_/2 MLBs, so *z*_1_ and *z*_2_ will have at least 2 ⋅ (*s*_*m*_/2)=*s*_*m*_ MLBs (unless both are in the AOI). Thus, both *z*_1_ and *z*_2_ are converged iterates. *z*_1_ can therefore be declared the root and *z*_2_ need not be calculated.

Condition *s*_1_≥*s*_*m*_/2 is insufficient when the start value *z*_0_ is zero. As an example, consider a double-precision iteration toward the root 10^−8^, starting from *z*_0_=0. If the first iterate is *z*_1_=2^−27^≃7.45×10^−9^ then, in accordance with Nikolajsen [14], $s_{1}=-\log_{2}(z_{1})=27$, so *s*_1_≥*s*_*m*_/2 is satisfied (since *s*_*m*_=53 for double-precision), so *z*_1_=7.45×10^−9^ will wrongly be declared the root. This can happen only when the start value *z*_0_ is zero, in which case the number of MLBs of *z*_0_ and *z*_1_ becomes $s_{1}=-\log_{2}(z_{1})$. It can therefore be avoided by disallowing zero start values. An alternative option, adopted here, is to switch to condition *s*_1_≥*s*_*m*_ when *z*_0_ is zero. *s*_1_≥*s*_*m*_ will always correctly indicate a root since it cannot be satisfied unless iterate *z*_1_ is exactly equal to start value *z*_0_ (*s*_*m*_ being the bit length of the floating-point significand used).

#### 4.3.2 Stopping criterion #3.2

$$z_{i}\text{ is a root if }(1)\;s_{i}-s_{i-1}\geq s_{m}/2\text{ or if }(2)\;s_{i}-s_{i-1}\geq s_{m}/4\text{ and }s_{i+1}-s_{i}<s_{i}-s_{i-1}\cdot \;i\geq 2\text{ required.}$$

Part (1) of stopping criterion #3.2 states that when convergence is so rapid that the number of MLBs gained in a single iteration (#*i*) is greater than or equal to half the number of bits of the floating-point significand then iterate *z*_*i*_ cannot be improved further and can be declared a root. Such rapid convergence is easily fast enough to ensure that it will not diminish until the AOI has been reached. Therefore, if iteration #(*i*+1) is carried out, it will also produce a gain of at least *s*_*m*_/2 MLBs, unless the AOI is reached. *z*_*i*_ and*z*_*i*+1_ will therefore have at least 2⋅(*s*_*m*_/2)=*s*_*m*_ MLBs, which is the maximum possible, or they will both reside in the AOI. *z*_*i*_ and *z*_*i*+1_ will therefore both be converged iterates, so *z*_*i*_ can be declared a root and *z*_*i*+1_ need not be calculated. No preconditions are needed because a gain of *s*_*m*_/2 MLBs in a single iteration is so large that it leaves no doubt that convergence has started.

Part (2) of the stopping criterion deals with occasions when convergence is rapid enough so that between *s*_*m*_/4 and *s*_*m*_/2 MLBs are gained in a single iteration (#*i*), in other words, *s*_*m*_/4<*s*_*i*_−*s*_*i*−1_<*s*_*m*_/2, in which case part (1) of the stopping criterion is not satisfied. Experience shows that a gain of at least *s*_*m*_/4 MLBs in a single iteration is sufficient to ensure that the rate of convergence does not diminish until the AOI has been reached. So if the rate of convergence does diminish, i.e. if *s*_*i*+1_−*s*_*i*_<*s*_*i*_−*s*_*i*−1_, then both *z*_*i*_ and *z*_*i*+1_ must reside in the AOI of the root. *z*_*i*_ can therefore be declared a root and *z*_*i*+1_ need not be calculated.

With stopping criterion #3.2 in place, stopping criterion #3.1 looks almost redundant, being invoked only in the small number of cases when the start value hits a root. However, neither stopping criterion #1, #2 nor #3.2 will identify such a root, so several redundant iterations will be required before the root is eventually identified by stopping criterion #4 (described next).

### 4.4 Stopping criterion #4

$$z_{i+1}\text{ is a root if }s_{i+2}\leq s_{i+1}.\text{ Preconditions: }s_{i-1}\geq b,s_{i}\geq b\text{ and }s_{i+1}\geq s_{i}.\;b=8\text{ is recommended.}$$

Stopping criterion #4 acts as a safety net designed to catch roots which are too ill-conditioned, or whose convergence rate is too slow, to invoke the previous stopping criteria. Stopping criterion #4 is wholly empirical and is a combination of the following two sub-criteria:

Sub-criterion #1: *z*_*i*+1_ *is a root if* *s*_*i*−1_≥*b*, *s*_*i*_≥*s*_*i*−1_, *s*_*i*+1_≥*s*_*i*_, and *s*_*i*+2_≤*s*_*i*+1_. This loosely states that once convergence has started and has been sustained over at least two consecutive iterations (as reflected by *s*_*i*−1_≥*b*, *s*_*i*_≥*s*_*i*−1_ and *s*_*i*+1_≥*s*_*i*_) then a failure to gain additional MLBs in the following iteration (as reflected by *s*_*i*+2_≤*s*_*i*+1_) indicates that the AOI has been reached, so a root can be declared.

Sub-criterion #2: *z*_*i*+1_ *is a root if* *s*_*i*−1_≥*b*, *s*_*i*_≥*b*, *s*_*i*+1_≥*b*, *s*_*i*_≤*s*_*i*−1_, *s*_*i*+1_≥*s*_*i*_ and *s*_*i*+2_≤*s*_*i*+1_. This is based on the observation that once at least *b* MLBs have been achieved in three consecutive iterations then alternate loss and gain of MLBs over the same three iterations indicates that no additional MLBs can be extracted, so a root can be declared.

Inspection of the two sub-criteria shows that they are identical except for *s*_*i*_≥*s*_*i*−1_ in the first and *s*_*i*_≤*s*_*i*−1_ in the second. They can therefore be merged into a single criterion (i.e. stopping criterion #4) with no restriction on the relative values of *s*_*i*_ and *s*_*i*−1_. Note also that when the sub-criteria are merged, condition *s*_*i*+1_≥*b* in sub-criterion #2 becomes redundant.

Stopping criterion #4 works on the same basic principle as Ward's criterion, discussed in §3. Like Ward's, it requires at least one and possibly several post-convergence iterations with the final calculated iterate being *z*_*i*+2_, which is needed to determine *s*_*i*+2_. Thus, at least *z*_*i*+2_ and *z*_*i*+1_ (and possibly several additional previous iterates) will be in the AOI of the root. *z*_*i*+1_ is chosen as the root because experience shows that it is often slightly more accurate than *z*_*i*+2_.

Note that preconditions *s*_*i*−1_≥*b* and *s*_*i*_≥*b* (with *b*=8) are stricter than precondition $s_{i-1}\geq s_{m}/q_{m}^{2}≃5.89$ for stopping criteria #1 and #2. This is necessary because, unlike stopping criteria #1 and #2, stopping criterion #4 (*s*_*i*+2_≤*s*_*i*+1_) does not in itself contribute to ensuring that convergence has started.

*b* must be kept as small as possible because roots with less than *b* significant leading bits will be missed by stopping criterion #4. The suggested *b*-value of 8 is the smallest that prevents premature termination for all the test cases run in §7. With *b*=8, roots with less than 8 ⋅ *log*_10_2≃2.4 significant decimal digits will be missed. (Inspection of Ward's criterion shows that it will miss roots with less than 3 significant decimal digits.) Although roots with less than 2.4 significant decimal digits are relatively rare in most applications, their existence does leave room for further improvement of stopping criterion #4. Still, it is remarkable that a stopping criterion as simple as #4 exists, which has not been observed to miss a root when *b*=8.

### 4.5 General remark

The same iteration formula must be used to calculate all the *s*-values required in all the stopping criteria presented in §4. Otherwise, one of the stopping criteria may be invoked prematurely by a change in convergence rate caused by a switch of iteration formula.

## 5. Zero roots

It has so far been assumed that a non-zero, real root is being approached. When the approach is toward a zero root, *s*_*i*_ (the number of leading bits that *z*_*i*_ has in common with *z*_*i*−1_) must be replaced by *s*_*oi*_ (the number of leading bits that *z*_*i*_ has in common with zero, i.e. the number of leading zero bits of *z*_*i*_). This is because, on approach to a zero root, it is the size of *z*_*i*_'s *exponent* that indicates its proximity to zero, whereas, the number of MLBs of *z*_*i*_ and *z*_*i*−1_'s *significands* is of little interest. In practice, it is not always possible to distinguish between an iteration sequence that approaches a near-zero root and one that approaches a zero root, so both *s*_*i*_ and *s*_*oi*_ must be calculated. A near-zero root is then indicated if the *s*_*i*_ sequence satisfies one of the stopping criteria of §4. And a zero root is indicated if *s*_*oi*_ satisfies one of the stopping criteria. The calculation procedure for *s*_*oi*_ is outlined in Nikolajsen [14].

One exception is stopping criterion #2, which should not be used to identify zero roots. Consider, for example, an iteration sequence starting at *z*_0_=1.000000×10^0^ and approaching the single-precision root *z*=5.555555×10^−6^. The iterates will typically show strong growth in *s*_*oi*_ as they move quickly down through the decimal exponents from 0 to −6. But as exponent −6 is reached, the growth in *s*_*oi*_ will stall and be replaced by growth in *s*_*i*_ as the significand approaches 5.555555. However, the growth in *s*_*oi*_ can on rare occasions be so strong that it invokes stopping criterion #2 prematurely, falsely indicating that the root is zero.

An alternative way of finding a zero root is simply to use a zero start value. If a zero root exists, the iterates will be so close to zero that stopping criterion #3 will likely be invoked within one or two iterations. But that only works when deflation is used, i.e. for polynomials. If root suppression is used (as with matrices and general nonlinear functions) a zero start value, coinciding with a zero root already extracted, will likely cause a floating-point exception, unless another zero root exists.

## 6. Complex roots

The stopping criteria in §4 can also be used to terminate the iterations toward a complex root *z*=*x*+*jy*. A procedure for calculating *S*_*i*_ (the number of MLBs for complex iterates) is outlined in appendix A.

It is tempting to try to avoid using *S*_*i*_ by using *s*_*i*_ instead to check the real and imaginary iterations streams, *x*_*i*_ and *y*_*i*_, separately for convergence. But experience shows that can go wrong in rare instances, for example, when the complex iterates *z*_*i*_ make a move almost parallel to the *x*-axis, resulting in *y*_*i*_≃*y*_*i*−1_, followed by a move almost parallel to the *y*-axis, resulting in *x*_*i*+1_≃*x*_*i*_, before convergence has been completed. Inspection of stopping criteria #1 and #3 shows that they can be invoked prematurely by the resulting *s*_*i*_ sequences. This cannot be avoided by requiring simultaneous completion of convergence for both streams because (i) the two streams often do not complete their converge simultaneously and (ii) the stopping criteria are optimized to the extent that if completion of convergence is not accepted immediately when it occurs, it may not be triggered by subsequent iterations.

A review of appendix A will confirm that *S*_*i*_ sequences do not invoke any of the stopping criteria prematurely in the situation described above, so it can be used instead. But *S*_*i*_ is not sufficient: *s*_*oi*_ (as defined in §5) is also needed to check the *y*_*i*_ and *x*_*i*_ streams individually for convergence to zero, i.e. to check if *z*_*i*_ is approaching a real or an imaginary root. If the *s*_*oi*_ sequence for the *y*_*i*_ stream invokes a stopping criterion (indicating that a real root is being approached), then *y*_*i*_ and all subsequent *y*-iterates must be zeroed. Otherwise, the following iterations may provide additional, redundant improvements of *y*_*i*_, at the expense of little or no improvement in *x*_*i*_. At best, this delays convergence. At worst, the temporary lack of improvement in *x*_*i*_ can result in the *S*_*i*_ sequence stalling temporarily, thereby triggering one of the stopping criteria prematurely. The same applies on approach to an imaginary root.

The approach of a complex iteration sequence *z*_*i*_=*x*_*i*_+*jy*_*i*_ to a real root may also manifest itself by |*x*_*i*_|+|*y*_*i*_|=|*x*_*i*_| to within floating-point accuracy. In that case, *z*_*i*_=*x*_*i*_+*jy*_*i*_ should be replaced by *z*_*i*_=*x*_*i*_. Otherwise, experience shows that *y*_*i*_ may continue to approach zero monotonically for many redundant iterations after *x*_*i*_ has converged. The approach to an imaginary root should be treated similarly.

One exception to the above is that *S*_*i*_ should not be used with stopping criterion #4. Rare instances have been encountered where the first few iterates have been so closely spaced that *S*_*i*_ has invoked stopping criterion #4 before convergence has started. Inspection of the raw data suggests that these iterates are close because they are struggling to break free from the attraction of a nearby root that has already been found and suppressed, but whose accuracy is insufficient for the suppression to fully eliminate its attraction. Experience shows that this type of premature triggering of stopping criterion #4 by *S*_*i*_ can be avoided by using *s*_*i*_ instead for the *x*_*i*_ and *y*_*i*_ iteration streams separately and requiring stopping criterion #4 to be triggered for both before a root is declared. Triggering need not be simultaneous. One reason why this works is that, before convergence has started, the chance of the *x*_*i*_ and *y*_*i*_ iteration streams both inadvertently satisfying stopping criterion #4 is exceedingly small. Also, the requirement to use *S*_*i*_ instead of *s*_*i*_, as outlined above, does not apply to stopping criterion #4: inspection of that criterion shows that, unlike stopping criteria #1 and #3, it cannot be invoked by isolated occurrences of *y*_*i*_≃*y*_*i*−1_ and *x*_*i*+1_≃*x*_*i*_.

## 7. Comparison of stopping criteria

The new stopping criteria, outlined in §4, are compared numerically with those of Ward, Igarashi and Grant & Hitchins, which are reviewed in §3. For short, the criteria will be named JLN, Ward, Iga and G&H, respectively.

The stopping criteria are compared in terms of efficiency and accuracy. Efficiency is measured simply in terms of the number of function evaluations required before a root is declared. Accuracy is measured in terms of the number of fractional significant digits (FSDs) of the extracted roots relative to the exact roots whenever these are available. When they are not, other similar accuracy measurements will be used as specified in each case.

All the results are based on IEEE quad-precision calculations. All the calculations were repeated in double-precision. The double-precision results led to the same general conclusions as the quad-precision results. Single-precision testing was omitted as being of little practical interest, given the current state of the art in computer hardware.

### 7.1 Numerical implementation

The stopping criteria are all embedded in the same versions of Laguerre's and Ostrowski's methods, both of which are third order methods described for example in Orchard [4].

Laguerre's method is used for matrix eigenvalue extraction and polynomial root finding. Ostrowski's method is used for general nonlinear functions. Deflation is used for polynomials and root suppression is used for matrices and general nonlinear functions to prevent repeated convergence to the same root. Both Laguerre and Ostrowski lend themselves to the same efficient root suppression procedure, as outlined for example in Nikolajsen [13]. The simplicity and reliability of Laguerre and Ostrowski allow the focus of the comparisons to remain on the stopping criteria. Using the same iteration procedures throughout also ensures that identical iteration streams are generated regardless of which set of stopping criteria is used. Thus, the stopping criteria affect the results only by deciding which iterate in the iteration stream is declared a root.

In practice, the stopping criteria do have a small effect on the iteration streams for the following reasons. (i) When different iterates are declared the root, root suppression and deflation give rise to small differences in the remaining function, and thus in the size of the remaining roots, and thus possibly in the number of iterations needed to extract them. (ii) The start value for each iteration is chosen as the root just found (when deflation is used) and as a non-converged iterate toward a previous root (when root suppression is used). This can likewise result in small differences in the remaining iterations streams, and thus possibly in the number of iterations needed to find the remaining roots. But, as will be seen, the test conclusions are so unambiguous that they are unaffected by a tolerance of plus or minus a few iterations.

For matrix eigenvalue extraction, only JLN's and Ward's criteria are applicable and will be compared. For polynomial root extraction, both JLN, Ward, Iga and G&H are applicable and will be compared. For non-polynomial root extraction, JLN, Ward and Iga are applicable and will be compared.

The JLN stopping criteria are implemented using the continuous FSD formulation given in Nikolajsen [14]. The criteria are applied in the following order of descending efficiency: #3, #1, #2 and #4. The order of application affects the percentage usage of the criteria because two criteria are often satisfied simultaneously.

Ward's stopping criterion *e*_*i*_>*e*_*i*−1_ is replaced by *e*_*i*_≥*e*_*i*−1_ because *e*_*i*_>*e*_*i*−1_ fails to activate when *e*_*i*_ approaches zero monotonically and then remains equal to zero. For complex root finding, Ward's criterion is applied to the real and imaginary iteration streams separately because Ward was found in one instance to be triggered prematurely when applied directly to the complex iteration stream.

G&H's stopping criterion is supplemented by the following criterion: *z*_*i*+1_ *is a root if z*_*i*+1_=*z*_*i*_. Without this, G&H does not get invoked when the start value by chance hits a root.

### 7.2 Matrix eigenvalue extraction

**Figure 1.**
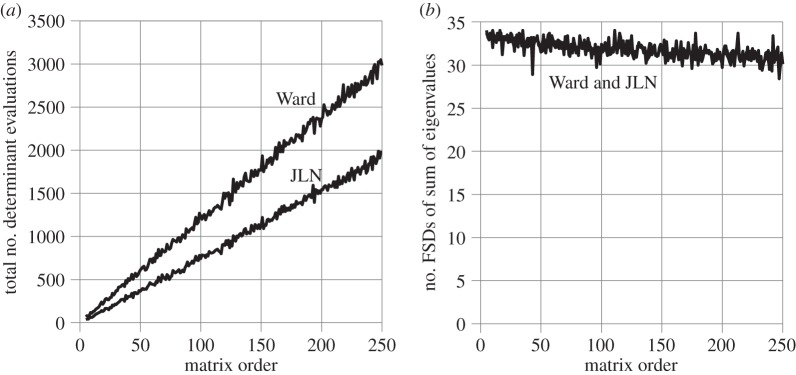
Dense, random matrices.

Figure 1*a* shows the total number of determinant evaluations needed to find all the eigenvalues of dense, random, non-symmetrical matrices of orders 5 through 250 using JLN's and Ward's stopping criteria. The same random matrices are used for both. The matrices are reduced to upper Hessenberg form to allow the eigenvalues to be extracted by Laguerre's method, as outlined in Nikolajsen [13]. When a complex eigenvalue is found, its complex conjugate is also declared an eigenvalue.

Figure 1*b* shows the corresponding accuracy of Ward and JLN, expressed in terms of the number of FSDs of the sum of eigenvalues relative to the matrix trace. The sum of eigenvalues is used because the exact eigenvalues are unknown. This is considered to be acceptable here because the matrix elements are all in the range −1 to 1, resulting in similar sized eigenvalues, so no single eigenvalue will dominate the sum. The JLN and Ward lines in figure 1*b* almost coincide. Thus, JLN and Ward provide practically the same accuracy for the random matrices used here. The average number of FSDs achieved across the entire graph is 31.8 with both JLN and Ward out of a maximum possible 34. The small overall slope of the graphs in figure 1*b* indicates that the matrix order has little effect on accuracy. This confirms the common observation that random matrices, of the type used here, are very well-conditioned. The jitter in all the graphs in figure 1 confirms that the matrices are not all exactly equally well-conditioned.

The total number of eigenvalues extracted is 5+6+⋯+250=31 365 and the total number of determinant evaluations is 375 447 with Ward and 238 416 with JLN. Thus, the average number of determinant evaluations per eigenvalue is 375 447/31 365=11.97 with Ward and 238 416/31 365=7.60 with JLN. JLN therefore reduces the number of determinant evaluations per eigenvalue by an average of 4.37 or 4.37/11.97=36.5% without compromising the accuracy of the results. The relatively low level of jitter in figure 1*a* confirms that these averages do not hide any major discrepancies in the number of determinant evaluations needed to extract the eigenvalues of each matrix.

The percentage usage of JLN stopping criteria #1, #2, #3 and #4 is 69.3%, 0%, 30.7% and 0%, respectively. In other words, Laguerre's method converges so fast for these types of matrices that a stopping criterion as demanding as #3 gets invoked for 30.7% of the eigenvalues, whereas the two slowest and least demanding criteria (#2 and #4) do not get invoked at all.

**Figure 2.**
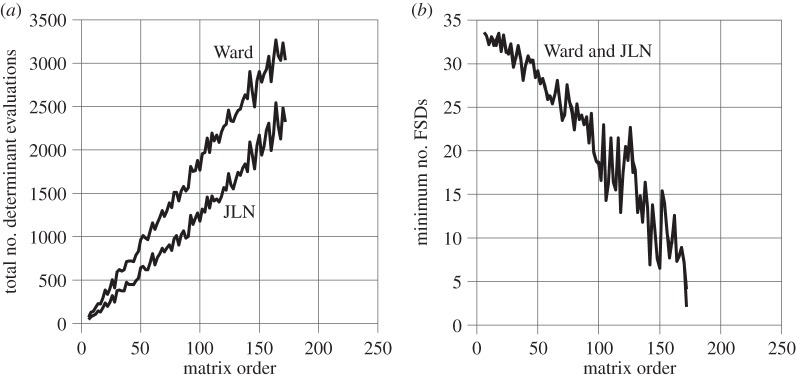
Dense, random Hessenberg matrices.

Figure 2 shows the same type of results as figure 1 but for more ill-conditioned matrices, i.e. random lower Hessenberg matrices with 2×2 bulges along the diagonal, which allow the exact eigenvalues to be calculated *a priori*. The matrices are reduced to *upper* Hessenberg form before eigensolution. The accuracy is shown in figure 2*b* in terms of the number of FSDs of the least accurate eigenvalue of each matrix relative to its exact counterpart. The ill-conditioning causes the graphs to terminate at matrix order 172, at which point the accuracy becomes so poor that the least accurate eigenvalue can no longer be matched unambiguously with its exact counterpart. This is reflected in the graphs in figure 2*b* approaching zero. Note also the greater overall slopes in figure 2*a*, compared with figure 1*a*, due to the larger number of iterations needed to find the eigenvalues.

The JLN and Ward lines in figure 2*b* almost coincide. Thus, JLN and Ward provide practically the same accuracy for the matrices used here. The average number of FSDs achieved for the least accurate eigenvalue of each matrix is 21.7 with both JLN and Ward out of a maximum possible 34. The steep overall slopes in figure 2*b* reflect a rapid deterioration in the accuracy of the eigenvalues with increasing matrix order. The strong accompanying jitter suggests a significant variation in the level of ill-conditioning of the matrices.

The total number of eigenvalues extracted is 6+8+⋯+172=7476 and the total number of determinant evaluations is 138 549 with Ward and 95 496 with JLN. Thus, the average number of determinant evaluations per eigenvalue is 138 549/7476=18.53 with Ward and 95 496/7476=12.77 with JLN. Thus, JLN reduces the number of determinant evaluations per eigenvalue by an average of 5.76 or 5.76/18.53=31.1% without compromising the accuracy of the results. This is a larger absolute savings but a smaller percentage savings compared with the matrices of figure 1. The percentage savings is smaller only because the number of function evaluations per eigenvalue is larger.

The percentage usages of JLN stopping criteria #1, #2, #3 and #4 are 71.6%, 10.6%, 16.7% and 1.0%, respectively. Thus, the ill-conditioning has forced down the use of stopping criterion #3 and created a demand for stopping criteria #2 and #4, which were not used at all for the matrices of figure 1.

Thus, the advantage in efficiency of JLN over Ward has been demonstrated for both well-conditioned and ill-conditioned matrices. The almost equal accuracy of JLN and Ward, and the superior efficiency of JLN, extends over all the test cases run, which include a total of approximately 97 227 eigenvalue extractions for approximately 829 matrices of different sizes and different levels of ill-conditioning. Results are shown in the electronic supplementary material.

Testing with very ill-conditioned matrices was omitted. With such matrices, one has to be thankful just to be able to extract the eigenvalues, let alone doing so with high efficiency.

### 7.3 Polynomial root finding

Jenkins & Traub [15] suggest that stopping criteria for polynomial root finding be tested on the following type of polynomials:

$$p(z)=b(z-r)(z+r)(z-1)=b(z^{3}-z^{2}-r^{2}z+r^{2}),$$

with both *r* and *b* numerically large and small. The purpose is to check whether termination problems are caused by numerically large and/or small roots and numerically large and/or small polynomial coefficients. In the current test series, large variations in both *r* and *b* are effected by using polynomials of the type

$$p_{1}(z)=\prod_{r=\pm 1}^{\pm n/4}(z\pm (2^{r}+j2^{r})),$$

where *n* is increased in integer multiples of 4 from 8 to 256. The roots with the numerically largest and smallest moduli are 2^64^+*j*2^64^≃1.8×10^19^+*j*1.8×10^19^ and 2^−64^+*j*2^−64^≃5.4×10^−20^+*j*5.4×10^−20^. The corresponding numerically largest and smallest real and imaginary parts of the polynomial coefficients are approximately 3.3×10^635^ and 8.4×10^−637^.

Jenkins & Traub [15] also suggest testing the stopping criteria on Wilkinson-type polynomials:

$$p(z)=\prod_{r=1}^{n}(z-r),$$

with *n* small enough to ensure exact representation of all the polynomial coefficients at the precision level used. The objective is to test whether the extreme ill-conditioning of such polynomials cause termination problems. In the current tests, this is extended into the complex domain by using the following similarly ill-conditioned polynomials:

$$p_{2}(z)=\prod_{r=1}^{n}(z-(r+j\cdot r)).$$

Finally, Jenkins & Traub [15] suggest that stopping criteria based on round-off error analysis should be tested on polynomials of the type

$$p(z)=\prod_{r=1}^{n}(z-10^{-r}),$$

with *n* small enough to avoid underflow of the polynomial coefficient. In the current test series, the *p*_1_(*z*) polynomials, defined above, are used for this purpose.

The polynomial coefficients for both *p*_1_(*z*) and *p*_2_(*z*) are calculated numerically based on the known exact roots, whereafter, the approximate roots are extracted. The coefficients are automatically scaled as they are calculated to delay overflow and underflow and thus maximize the range of calculable roots.

#### 7.3.1 Polynomials of type *p*_1_(*z*)

**Figure 3.**
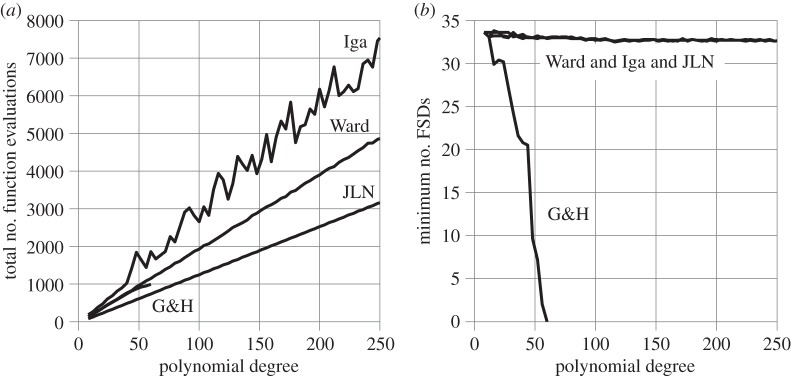
Polynomials of type *p*_1_(*z*).

Figure 3*a* shows the total number of function evaluations needed to find all the roots of polynomials of type *p*_1_(*z*) (defined above) of degree 8 through 250 using JLN's, Ward's, Igarashi's and Grant & Hitchins' stopping criteria. Figure 3*b* shows the accuracy of the least accurate root of each polynomial in terms of the number of FSDs relative to the corresponding exact root.

Note that G&H only manages to find the roots of polynomials up to degree 56, at which point the accuracy becomes so poor that the least accurate root can no longer be matched unambiguously with its exact counterpart. This is caused by G&H's tendency to terminate the iterations prematurely, one or two iterations before the most accurate root has been found. Each time one of the premature roots is deflated out, the remaining polynomial loses accuracy, leading to a chain reaction of root deterioration. The steepness of the G&H line in figure 3*b* indicates how quickly this happens. The current formulation of G&H's stopping criterion is therefore considered to be unacceptable for practical purposes.

Figure 3*b* shows that Ward, Iga and JLN retain almost equal, high accuracy throughout. But the high accuracy of Iga is deceptive. Inspection of the raw data shows that the sawtooth shape of the Iga line in figure 3*a* is caused by intermittent failures to trigger when a root has been found. Each failure allows the number of iterations to grow until the root finder eventually stops automatically when a preset limit of 64 iterations has been reached. The failures are distributed across almost the entire range of polynomial degrees, as evidenced by the sustained sawtooth shape of the Iga line in figure 3*a*. Each failure to trigger is of course also a failure to confirm that the final 64th iterate is a root. Iga fails for 70 of the 7808 roots found in figure 3, which is a failure rate of less than 1%. Nevertheless, the resulting loss of confidence is deemed to make Igarashi's stopping criterion unacceptable in its current formulation.

That leaves Ward and JLN as the only acceptable category (2) stopping criteria for the polynomials tested here. Ward and JLN achieve average FSD values of 32.9 and 32.8, respectively, for the least accurate root of each polynomial tested, making them equally accurate for all practical purposes. But JLN achieves this with 35.5% fewer function evaluations than Ward.

The percentage usages of JLN stopping criteria #1, #2, #3 and #4 are 46.3%, 0%, 53.7% and 0% respectively, i.e. the fastest and most demanding criterion (#3) gets invoked for more than half the roots, whereas the slowest and least demanding (#2 and #4) do not get invoked at all. This, once more, demonstrates the remarkable convergence speed of Laguerre's method and the ability of the JLN stopping criteria to take advantage of it.

#### 7.3.2 Polynomials of type *p*_2_(*z*)

**Figure 4.**
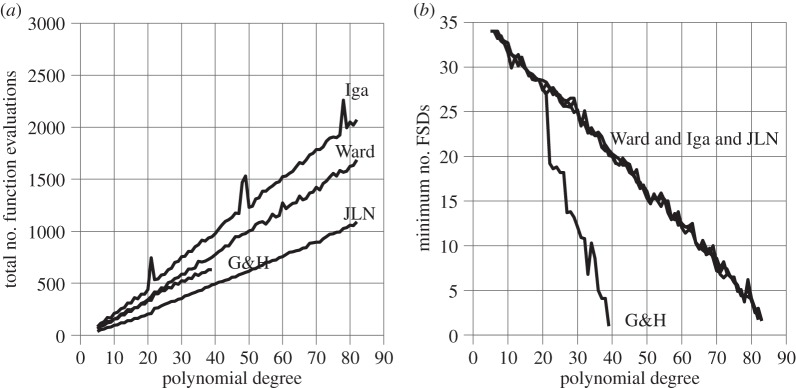
Polynomials of type *p*_2_(*z*).

Figure 4 shows the same type of results as figure 3 but for polynomials of type *p*_2_(*z*). The ill-conditioning of these polynomials causes the graphs to terminate at polynomial degree 82, at which point the accuracy becomes so poor that the least accurate root can no longer be matched unambiguously with its exact counterpart. Ward, Iga and JLN manage to identify all the roots up to polynomial degree 82 with almost equal accuracy. But Iga's accuracy is again deceptive since Iga fails to terminate the iterations for four roots out of the total of 5+6+⋯+82=3393. The number of iterations for each of the missed roots reaches 64 before the iterations are stopped by the root finder. Three of the misses show up as peaks in figure 4*a*. G&H only manages to match the accuracy of Ward and JLN up to a polynomial degree of 21 and fails completely at degree 38. These results support the decision in the previous section to reject Iga and G&H.

Thus, Ward and JLN are again the only acceptable category (2) stopping criteria. Ward and JLN achieve average FSD values of 18.9 and 18.6, respectively, for the least accurate root of each polynomial tested, making them equally accurate for all practical purposes.

The total number of function evaluations is 66 942 with Ward and 42 096 with JLN; thus, the average number of function evaluations per root is 66 942/3393=19.73 with Ward and 42 096/3393=12.41 with JLN. JLN therefore reduces the number of function evaluations per eigenvalue by an average of 7.32 or 7.32/19.73=37.1%.

The percentage usages of JLN stopping criteria #1, #2, #3 and #4 are 74.3%, 0.5%, 25.1% and 0%, respectively. Thus, almost all the roots get identified by the two most efficient stopping criteria, #1 and #3. But #2 is now needed and the use of #3 has been forced down from more than one-half to about one-quarter of the roots compared with the polynomials of type *p*_1_(*z*). Still, the extreme ill-conditioning of *p*_2_(*z*)-type polynomials manifests itself not so much by increased difficulty in calculating the roots but rather by the rapid deterioration in the accuracy of the calculated roots. This ill-conditioning normally limits the polynomial degree, at which all the roots can be calculated, to about 40 for quad-precision calculations. Here, it is extended to 82 by cognizant scaling the polynomial coefficients at each stage of their calculation.

In conclusion, the polynomial tests reported in §7.3.1 and §7.3.2 lead to the same conclusions as the matrix tests of §7.2: JLN's and Ward's stopping criteria provide equally accurate results but JLN reduces the number of function evaluations by roughly one third. Igarashi's and G&H's stopping criteria are unsafe and must be rejected in their current formulations.

#### 7.3.3 Polynomials of type [*p*_2_(*z*)]^2^

**Figure 5.**
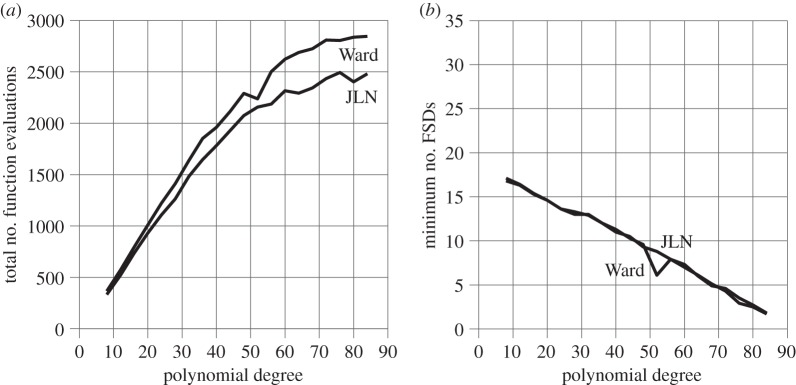
Polynomials of type [*p*_2_(*z*)]^2^.

In the previous polynomial examples, convergence is so rapid that stopping criteria #2 and #4 have hardly been used. Their use is demonstrated here by calculating the roots of polynomial type [*p*_2_(*z*)]^2^. All the roots are double roots and the single root Laguerre formula is used to slow the convergence rate to linear for half the roots in order force stopping #2 and #4 into action. The results are shown in figure 5. (Iga and G&H have been omitted because they have already been declared unacceptable.)

The percentage usage of JLN stopping criteria #1, #2, #3 and #4 changes from 74.3%, 0.5%, 25.1% and 0%, respectively, for *p*_2_(*z*)-type polynomials to 47.5%, 18.5%, 14.8% and 19.2% for [*p*_2_(*z*)]^2^-type polynomials. The 19.2% usage of stopping criterion #4 is still surprisingly low. The raw data suggest the following explanation. The lower accuracy of double roots accelerates the loss of accuracy of the deflated polynomials. As a result, the double roots still to be extracted gradually lose accuracy and split apart to become single roots, whose convergence rate is much greater. As a result, the faster stopping criteria, #1, #2 and #3, gradually take over from #4. This is also reflected in figure 5*a* by the progressive levelling off of both the Ward and the JLN lines as the number of function evaluations per root drops due to the greater speed of convergence of the emerging single roots.

A corresponding reduction might be expected in the maximum polynomial degree at which all the roots can be matched unambiguously to their exact counterparts. But comparison of figures 5*b* and 4*b* shows that this remains at 82. The reason appears to be that, once the double roots have split, any further deterioration in their accuracy is greatly reduced because they have become single roots that can be extracted with twice the number of MLBs. Of course, the first roots extracted will still be double roots, so the maximum accuracy cannot exceed half the single root accuracy, as also indicated by comparing figures 5*b* and 4*b*.

The coincidence of the JLN and Ward graphs in figure 5*b* again confirms that both are equally accurate. The exception is the blip in the Ward graph at polynomial degree 52. The raw data suggest that this is due to a small variation in the linear rate at which MLBs are gained when the first of a pair of double roots is extracted by the single root Laguerre formula. In this particular case, a very slow constant gain of 1.8 bits per iteration is interrupted by a premature loss of 0.6 bits, which triggers Ward's stopping criterion prematurely, reducing the number of correct decimal digits of the 21st root from 12.5 to 11.7 (also reducing the number of function evaluations, as shown in figure 5*a*). As this small error propagates through the subsequent deflations, it gradually reduces the accuracy of the subsequent roots to the point where the number of correct decimal digits of the poorest root drops from 8.8 to 6.1, as indicated in figure 5*b*. This could be avoided by putting a lower limit on the loss needed to trigger Ward, but that would reintroduce the problem which was solved in §7.1 by replacing *e*_*i*_>*e*_*i*−1_ by *e*_*i*_≥*e*_*i*−1_.

JLN stopping criterion #4 avoids this failure only by chance, i.e. because tiny differences between the iterates (caused by faster termination of previous roots) happen to produce a Laguerre iteration sequence that does not include a tiny premature loss of digits. This potential problem could be avoided by replacing *s*_*i*+2_≤*s*_*i*+1_ by *s*_*i*+2_≤*s*_*i*+1_−1 in stopping criterion #4. But that would lead to triggering difficulties in many other cases. The conclusion is that neither JLN stopping criterion #4 nor Ward is fail-safe at extremely slow convergence rates that can occur when the single root Laguerre formula is applied to a double root. But this is unlikely to happen in practice, when a sustained linear rate of convergence would be used to trigger a switch to the double-root Laguerre formula.

The total number of function evaluations is 39 288 with Ward and 34 905 with JLN. The total number of roots found is 8+12+16+⋯+84=920. Thus, the average number of function evaluations per root is 39 288/920=42.70 with Ward and 34 905/920=37.94 with JLN. Thus, JLN reduces the number of function evaluations per eigenvalue by an average of 4.76, compared with 7.32 for *p*_2_(*z*). In other words, the advantage of JLN over Ward, in terms of efficiency, is reduced from 37.1% for *p*_2_(*z*)-type polynomials to 4.76/42.70=11.1% for [*p*_2_(*z*)]^2^-type polynomials. The main reason is the more frequent need for JLN stopping criterion #4, whose efficiency is no better than Ward's. The reduced advantage of JLN is also evident from the closer proximity between Ward and JLN in figure 5*a* compared with figure 4*a*.

Polynomials with several other distributions of known roots were also tested, as were polynomials with random roots, for a total of approximately 739 polynomials of different degrees and levels of ill-conditioning with a total of approximately 60 227 roots. All the tests confirm the findings of this and the previous sections. Results are shown in the electronic supplementary material.

### 7.4 Root finding for general nonlinear functions

Igarashi's, Ward's and JLN's stopping criteria are implemented in a general nonlinear root finder based on Ostrowski's square root iteration formula in the complex domain (e.g. [4]). (‘Igarashi’ now refers to the criterion outlined in §3.2 with *W*=0.01 for transcendental functions and *W*=0.5 or *W*=1.0 for algebraic functions.)

Results are presented for the following sample functions:

$$\begin{array}{rl} f_{1}(z) & =\text{sin}\;z,\\ f_{2}(z) & =2\left(\frac{1}{\tanh z}-\frac{1}{\tan z}\right)+\left(\frac{1}{\sinh z}-\frac{1}{\sin z}\right)\\ \text{and}\;f_{3}(z) & =-1+(\cosh z-96z^{-3}\sinh z)\cos z+96z^{-3}(\cosh z-48z^{-3}\sinh z)\sin z. \end{array}$$

*f*_1_ is so simple that its exact roots are known. *f*_2_(*z*)=0 is one of two purely transcendental frequency equations for a beam with three equal-length spans on four pinned supports [16]. *f*_3_(*z*)=0 is the frequency equation for lateral vibration of a beam with flexible, pinned supports at each end [17].

Igarashi prescribes the use of *W*=0.01 for purely transcendental functions like *f*_1_ and *f*_2_ but does not make it clear which *W*-value should be used for functions like *f*_3_, which has both algebraic and transcendental terms. A preliminary value of *W*=0.01 is used for *f*_3_ because it is the least stringent and therefore the most likely to trigger Igarashi. This is an attempt to avoid Igarashi's failures to stop when a root has been found, as experienced earlier for both matrices and polynomials.

**Figure 6.**
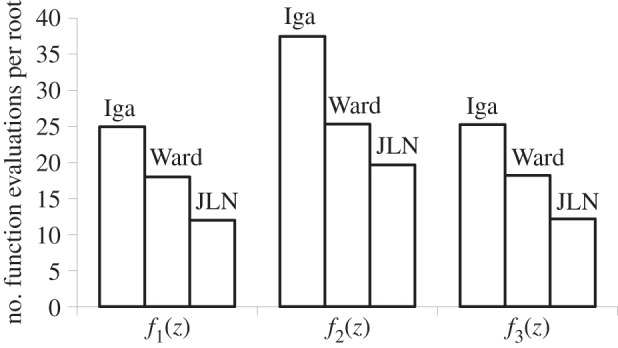
Average number of function evaluations per root.

Two hundred and fifty roots are extracted for both *f*_1_, *f*_2_ and *f*_3_. The average number of function evaluations per root are shown in figure 6. JLN's criteria can again be seen to be the most efficient, improving the efficiency by an average of 29.3% and 50.5% compared with Ward and Iga, respectively.

In terms of accuracy, Iga, Ward and JLN provide the exact same values for all 250 roots of both *f*_1_, *f*_2_ and *f*_3_. Inspection of the raw data shows that this is because (1) neither Iga, Ward nor JLN terminate the iterations until convergence has been completed and (2) in all cases, the first converged iterate and all subsequent iterates are exactly equal. No attempt was made to explain (2): the workings of the root solvers are beyond the scope of this paper. The important conclusion here is that all three sets of stopping criteria provide equal accuracy. Igarashi's previous failures to stop are not encountered with functions *f*_1_, *f*_2_ and *f*_3_.

Purely algebraic, non-polynomial functions are not common in practice but, for completeness, the roots of the following such functions are found:

$$f_{4}(z)=p_{1}(z)-z^{5/2}=\prod_{r=\pm 1}^{\pm n/4}(z\pm (2^{r}+j2^{r}))-z^{5/2}.$$

*p*_1_(*z*) is the polynomial tested in §7.3.1. The subtraction of *z*^5/2^ is used as a simple means of making *f*_4_ non-polynomial without introducing discontinuities in the *f*_4_-derivatives needed by Ostrowski. *f*_4_ has *n* roots, one for each of the *n* intersections between *p*_1_(*z*) and *z*^5/2^.

**Figure 7.**
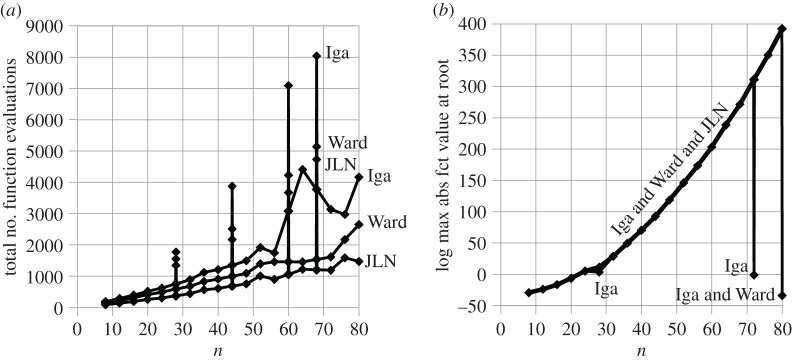
Nonlinear functions of type *f*_4_(*z*).

The results are shown in figure 7 for *n*=8,12,16,…80, with *W*=1.0 used for Igarashi. *f*_4_ turns out to be quite a challenge for Ostrowski's method, which fails to find the *n*th root for *n*=28, 44, 60 and 68, regardless of which set of stopping criteria is used. The four peaks in figure 7*a* are due to the additional iterations carried out in the fruitless search for the final root. The three topmost data points of each peak are the peak values for Iga, Ward and JLN, in the order shown for *n*=68.

The exact roots are unknown, so figure 7*b* gives the *relative* accuracy of the stopping criteria in terms of log_10_|( *f*_4_)|_max_, i.e. the logarithm of the largest absolute *f*_4_-value at the roots. The exact *f*_4_-values at the roots are of course zero. Any log_10_|( *f*_4_)|_max_-value less than −34 is set to −34 (the smallest achievable in quad-precision).

As shown in figure 7*b*, the accuracy of all three sets of stopping criteria is virtually the same except for (i) the tiny dip in the Iga line at *n*=28, (ii) the huge dips in the Iga line at *n*=72 and 80, and (iii) the huge dip in the Ward line at *n*=80. These dips do not signal any momentary improvement in accuracy. Rather, they occur for the following reasons:

(1) when Igarashi is applied, Ostrowski is only able to find 21 roots for *n*=28, 32 roots for *n*=72, and 35 roots for *n*=80,
(2) when Ward is applied, Ostrowski is only able to find 35 roots for *n*=80, and
(3) when so few of the *n* roots are found, log_10_|( *f*_4_)|_max_ becomes unrepresentatively small. This is because the roots are found roughly in ascending order of modulus, and the size of each log_10_|( *f*_4_)|-value is roughly proportional to the modulus of the corresponding root, so when only the small-modulus roots are found, the large log_10_|( *f*_4_)|-values are missing.

These problems appear to be related to relatively weak global convergence properties of Ostrowski's method. They are therefore beyond the scope of this paper and will not be pursued. It is also not clear why these problems are not encountered when JLN's stopping criteria are applied.

Inspection of the raw data shows that Iga fails to stop, when a root has been found, on a total of 25 occasions: once, twice, three times, 10 times and 11 times for *n*=28, 60, 64 and 72, respectively. With *W*=0.5 instead of 1.0, the total number of failures grows to 32. As before, the iterations are stopped automatically by the root finder, so the failures do not affect the apparent accuracy of Iga. But, as before, these failures are deemed to be unacceptable, thus disqualifying Igarashi's stopping criterion in its current form.

The average number of function evaluations per root is 12.391 with Ward and 9.070 with JLN for all the root extractions related to figure 7. Thus, in this case, JLN reduces the number of function evaluations per root by an average of 26.8% compared with Ward.

## 8. Summary

A new set of stopping criteria for iterative root finding and eigenvalue extraction has been presented, which terminates the iterations immediately when no further improvement of the results is possible. The new criteria, called JLN, have been tested numerically against the existing stopping criteria of Igarashi [3,5], Grant & Hitchins [7] and Ward [9]. The test results were as follows:

(1) Grant & Hitchins tended to trigger prematurely, leading to multiple failures. Igarashi occasionally failed to trigger, leading to multiple redundant iterations and uncertainty as to whether a root had been found. Both were therefore rejected in their current formulations.
(2) Ward and JLN were the only criteria that did not fail. Both provided the maximum possible accuracy of the results but JLN did so more efficiently, reducing the number of function evaluations by one-third without any deterioration in the accuracy of the results.
(3) Ward's criterion is by far the simplest and most easily implemented. So when simplicity is more important than efficiency, Ward's criterion is preferable. JLN's criteria are preferable in all other cases.

The range of testing reported here is of necessity limited and must be regarded as preliminary. But it is considered to be sufficiently convincing to warrant further consideration of the JLN stopping criteria when high efficiency is desired.

A numerical implementation of the JLN stopping criteria is available from the author on request.

## Supplementary Material

One file entitled: SupplementaryMaterial.pdf Contains additional figures supporting the findings outlined in the paper.

## Figures and Tables

**Figure 8.**
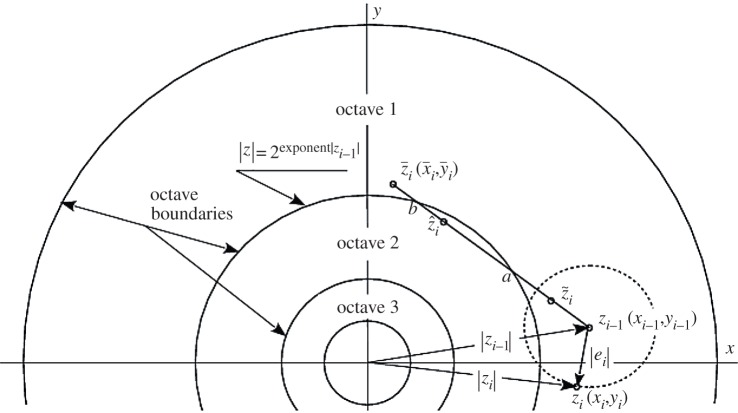
Snapshot of complex iterates *z*_*i*−1_ and *z*_*i*_.

## Appendix A. Matching leading bits for complex iterates

The number of matching leading bits (MLBs) of the complex iterates *z*_*i*−1_=*x*_*i*−1_+*jy*_*i*−1_ and *z*_*i*_=*x*_*i*_+*jy*_*i*_ is called *S*_*i*_. It is derived from the modulus $|e_{i}|=\sqrt{{\left(x_{i}-x_{i-1}\right)}^{2}+{\left(y_{i}-y_{i-1}\right)}^{2}}$ of the complex iteration increment *e*_*i*_=*z*_*i*_−*z*_*i*−1_=(*x*_*i*_−*x*_*i*−1_)+*j*(*y*_*i*_−*y*_*i*−1_) in the same way that *s*_*i*_ is derived from the increment |*e*_*i*_|=|*x*_*i*_−*x*_*i*−1_| of the real iterates *x*_*i*_ and *x*_*i*−1_ (see §4.1 and Nikolajsen [14]).

Figure 8 shows an example of corresponding values of $|z_{i-1}|=\sqrt{x_{i-1}^{2}+y_{i-1}^{2}},|z_{i}|=\sqrt{x_{i}^{2}+y_{i}^{2}}$ and $|e_{i}|=\sqrt{{\left(x_{i}-x_{i-1}\right)}^{2}+{\left(y_{i}-y_{i-1}\right)}^{2}}$. Rotation of |*e*_*i*_| about *z*_*i*−1_ demonstrates graphically that the same value of |*e*_*i*_| can result from differences in both length and direction of *z*_*i*−1_ and *z*_*i*_, confirming that *S*_*i*_ will reflect the differences in both modulus and argument of *z*_*i*−1_ and *z*_*i*_.

Just like |*e*_*i*_|=|*x*_*i*_−*x*_*i*−1_| must be corrected when *x*_*i*_ and *x*_*i*−1_ reside in different octaves, so the complex-based increment $|e_{i}|=\sqrt{{\left(x_{i}-x_{i-1}\right)}^{2}+{\left(y_{i}-y_{i-1}\right)}^{2}}$ must be corrected when it intersects an octave boundary. For complex numbers, an octave is defined as the annulus in the complex plane bounded by the circles |*z*|=2^*k*−1^ and |*z*|=2^*k*^, where *k* is an integer. This is a direct extension of the octave definition in Nikolajsen [14] for real numbers. In figure 8, octaves 1 and 2 are separated by the circle |*z*|=2^exponent|*z*_*i*−1_|^. With the octave boundary thus defined, *z*_*i*−1_ is located in octave 1 as shown. For simplicity, *z*_*i*−1_ and *z*_*i*_ are ordered such that |*z*_*i*−1_|≥|*z*_*i*_|. (*S*_*i*_ is unaffected by this ordering.) Thus, since *z*_*i*_ is closer to the origin, it will be located either in the same octave as *z*_*i*−1_ or in a lower octave. If *z*_*i*−1_ and *z*_*i*_ are neither in the same octave nor in adjacent octaves then they are so widely spaced that they have no MLBs in common, resulting in *S*_*i*_=0 (see Nikolajsen [14]). Thus, for *S*_*i*_ to be non-zero, *z*_*i*_ must be located either in octave 1 (*z*_*i*−1_'s octave) or in octave 2 (the next lower octave).

If line segment |*e*_*i*_|=*z*_*i*−1_*z*_*i*_ intersects the octave boundary |*z*|=2^exponent|*z*_*i*−1_|^ then |*e*_*i*_| must be adjusted to compensate for the fact that the distance between adjacent floating-point significand values in octave 1 is twice as large as in octave 2 (see Nikolajsen [14]). This is illustrated in figure 8 by assuming that point z_*i*_ is located first at ${\tilde{z}}_{i}$, then at ${\hat{z}}_{i}$, and finally at ${\bar{z}}_{i}$. If $z_{i}={\tilde{z}}_{i}$ then line segment |*e*_*i*_|=*z*_*i*−1_*z*_*i*_ does not intersect the octave boundary, so no correction of *e*_*i*_ is needed. If $z_{i}={\hat{z}}_{i},|e_{i}|=z_{i-1}{\hat{z}}_{i}$ intersects the boundary circle at point *a*, so |*e*_*i*_| must be replaced by $|e_{i}|+a{\hat{z}}_{i}$. If $z_{i}={\bar{z}}_{i},|e_{i}|=z_{i-1}{\bar{z}}_{i}$ intersects the boundary circle at points *a* and *b*, so |*e*_*i*_| must be replaced by |*e*_*i*_|+*ab*.

The length of the required line segment, $a{\hat{z}}_{i}$ or *ab*, can be found in the usual way based on the coordinates of points *a* and ${\hat{z}}_{i}$ or points *a* and *b*. The coordinates of points *a* and *b* are the solutions to the simultaneous equations for the line through points *z*_*i*−1_ and $\bar{z}$ or ${\hat{z}}_{i}$, and the boundary circle. If $z_{i}={\bar{z}}_{i}$, the parametric line equation becomes

A 1 $$\left\{\begin{array}{rl} x & =x_{i-1}+t({\bar{x}}_{i}-x_{i-1})\\ y & =y_{i-1}+t({\bar{y}}_{i}-y_{i-1}) \end{array}\right\}.$$

The circle equation is always

A 2 $$|z{|}^{2}=x^{2}+y^{2}=2^{2\cdot \mathrm{exponent}|z_{i-1}|}.$$

*a* and *b*'s coordinates (*x*,*y*) can be found by substituting equation (A 1) into equation (A 2) to find parameter *t*, then substituting *t* back into equation (A 1). The calculations are analogous when $z_{i}={\hat{z}}_{i}$.

If equations (A 1) and (A 2) have no solution (or one solution only) then line segment *z*_*i*−1_*z*_*i*_ does not intersect |*z*|=2^exponent|*z*_*i*−1_|^, so no |*e*_*i*_| adjustment is needed. If there are two solutions then *z*_*i*_'s octave can be identified as follows: if *z*_*i*−1_*z*_*i*_>*z*_*i*−1_*b* then *z*_*i*_ is located in *z*_*i*−1_'s octave and |*e*_*i*_| must be replaced by |*e*_*i*_|+*ab*. Otherwise, if *z*_*i*−1_*z*_*i*_>*z*_*i*−1_*a* then *z*_*i*_ is located in octave 2, so |*e*_*i*_| must be replaced by $e_{i}+a{\hat{z}}_{i}$. A numerical implementation is available from the author on request.

This procedure can be readily extended to finding the number of MLBs of two multi-dimensional iterates *z*_*i*−1_=(*z*_1_,*z*_2_,…,*z*_*n*_)_*i*−1_ and *z*_*i*_=(*z*_1_,*z*_2_,…,*z*_*n*_)_*i*_. But the resulting *S*_*i*_-values will be dominated by the larger components of *z*, so the procedure should only be used when *z* is truly a vector whose magnitude and direction are sought. If *z* is an array of equally important numbers then the number of MLBs should be calculated for each component individually.
