# Supplementary material for: New stopping criteria for iterative root finding
Source: R Soc Open Sci. 2014 Oct 15;1(2):140206. doi: 10.1098/rsos.140206 (PMC4448898; doi:10.1098/rsos.140206)
Supplement: One file entitled: SupplementaryMaterial.pdf Contains additional figures supporting the findings outlined in the paper. [file rsos140206supp1.pdf]

Supplementary Material

Nikolajsen, J.L. New stopping criteria for iterative root finding

<u>Nomenclature:</u>
Red line: Ward stopping criterion
Blue line: JLN stopping criteria
Lag: Laguerre's method
Ost: Ostrowski's method
Mat: Matrix eigensolution
Pol: Polynomial root extraction
128: Quadruple precision
64: Double precision
All axes labels are as in paper, also when not shown.
Column D, O, Z, AK refer to JLN, Ward, Igarashi, G&H respectively.
Column J, U, AF, AQ refer to JLN, Ward, Igarashi, G&H respectively.

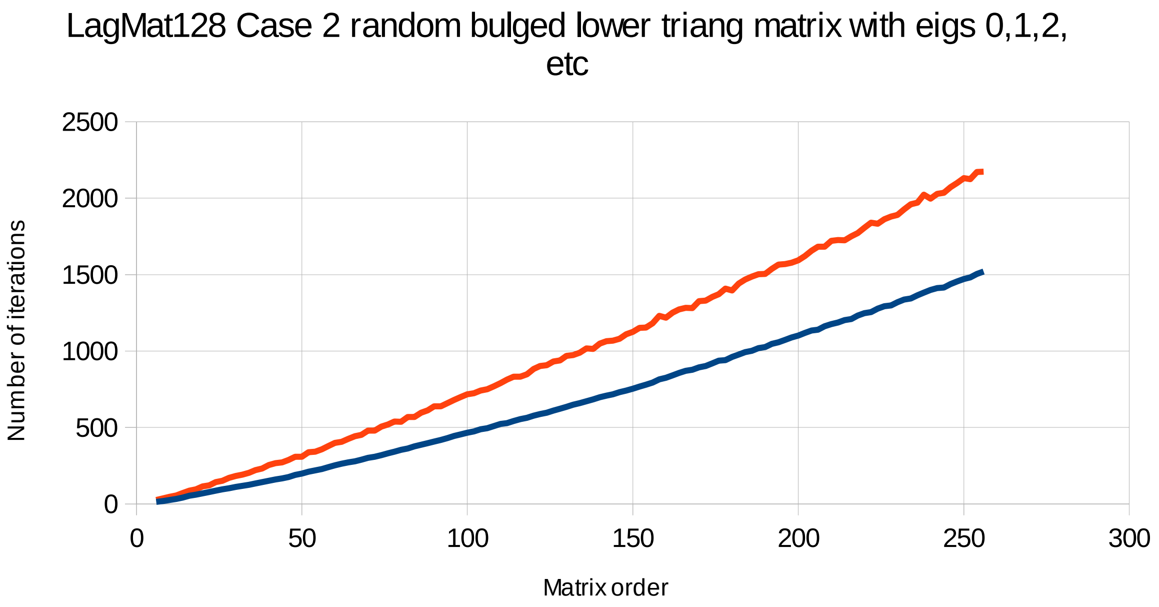

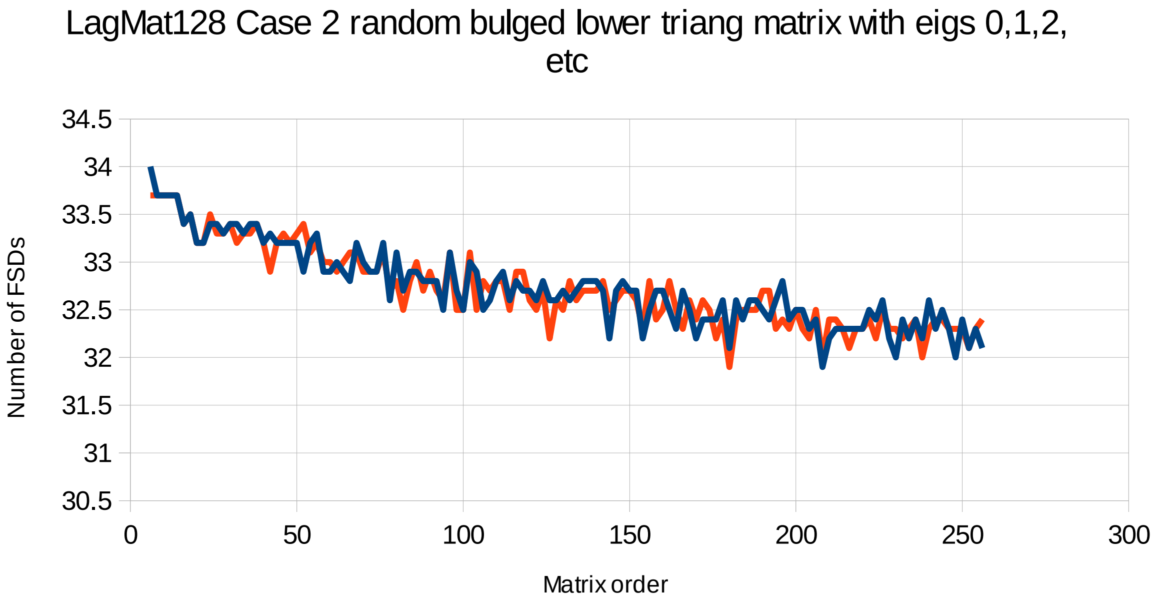

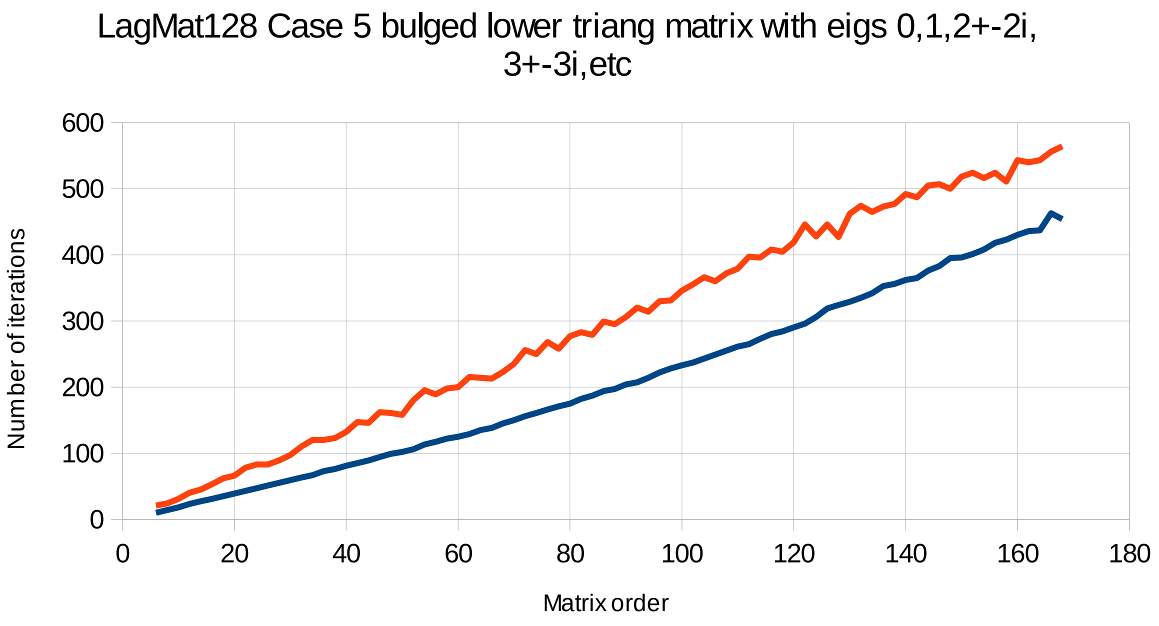
LagMat128 Case 5 bulged lower triang matrix with eigs 0,1,2+-2i, 3+-3i,etc
Number of iterations
Matrix order
0
100
200
300
400
500
600
0
20
40
60
80
100
120
140
160
180

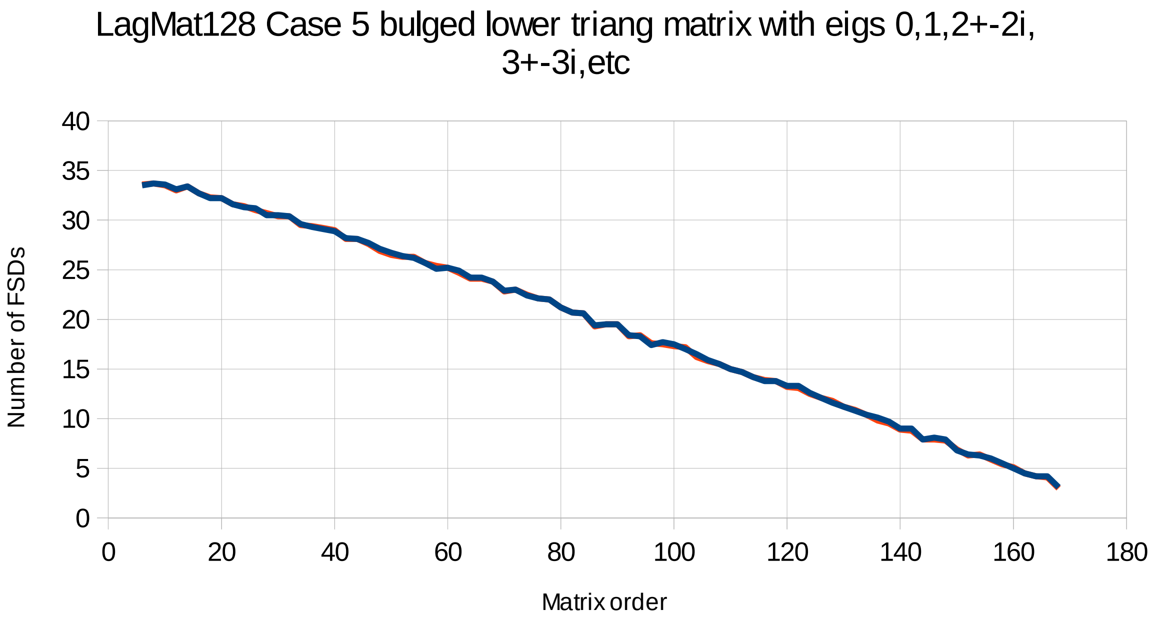
LagMat128 Case 5 bulged lower triang matrix with eigs 0,1,2+-2i, 3+-3i,etc
Number of FSDs
Matrix order
0
5
10
15
20
25
30
35
40
0
20
40
60
80
100
120
140
160
180

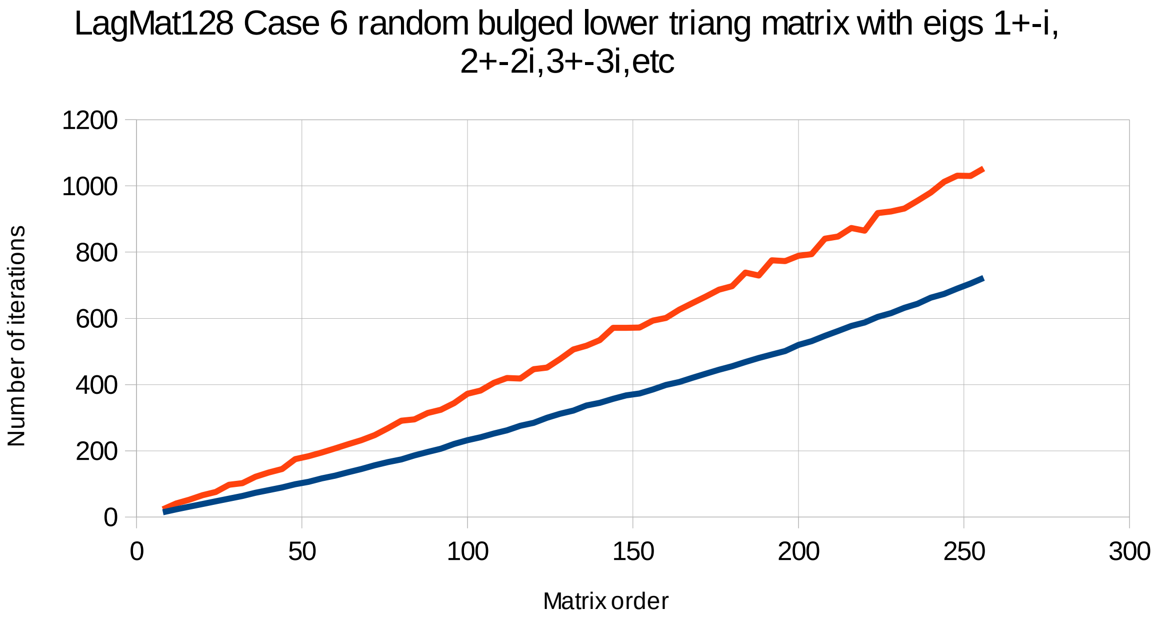
LagMat128 Case 6 random bulged lower triang matrix with eigs 1+-i, 2+-2i,3+-3i,etc
Number of iterations
Matrix order
0
200
400
600
800
1000
1200
0
50
100
150
200
250
300

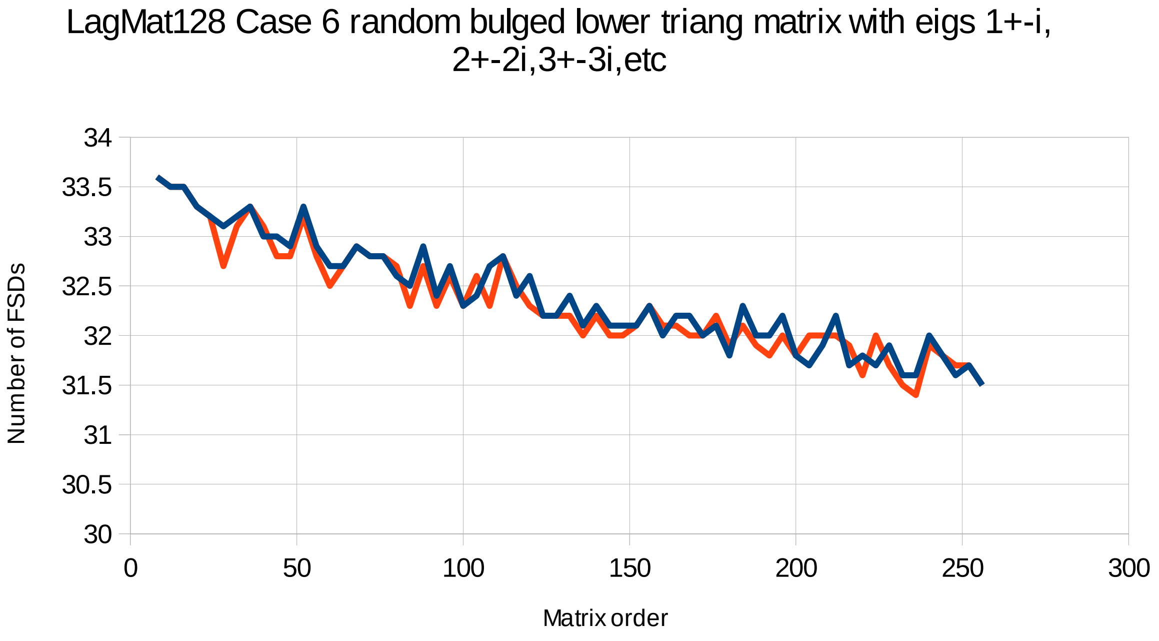
LagMat128 Case 6 random bulged lower triang matrix with eigs 1+-i, 2+-2i,3+-3i,etc
Number of FSDs
Matrix order
34
33.5
33
32.5
32
31.5
31
30.5
30
0
50
100
150
200
250
300

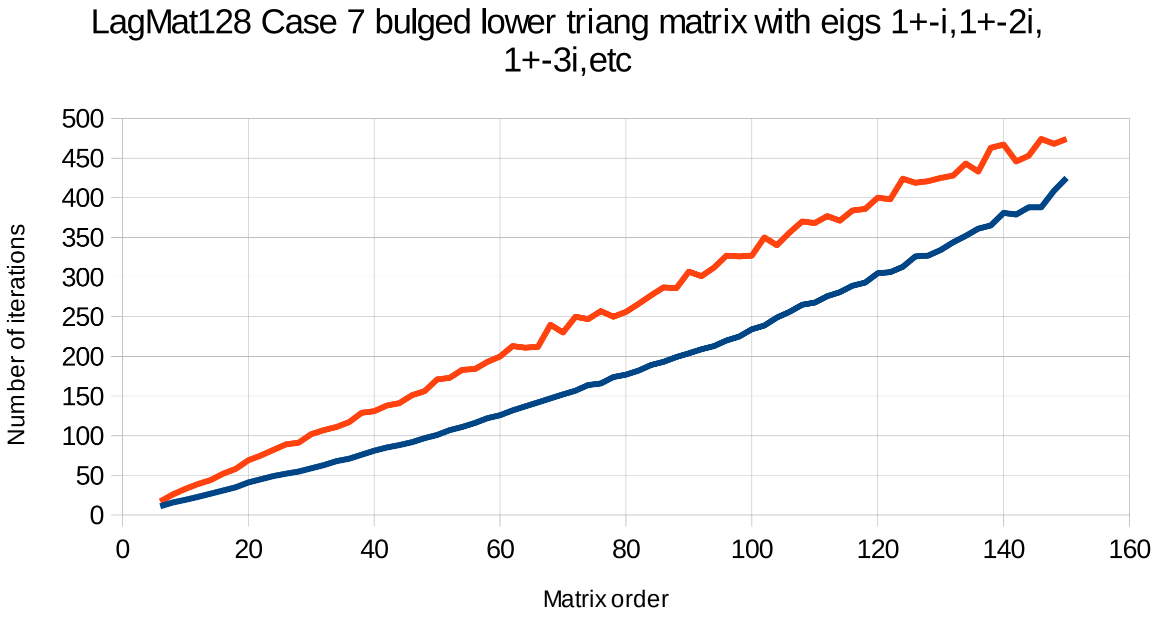
LagMat128 Case 7 bulged lower triang matrix with eigs 1+-i,1+-2i, 1+-3i,etc
Number of iterations
Matrix order
500
450
400
350
300
250
200
150
100
50
0
0
20
40
60
80
100
120
140
160

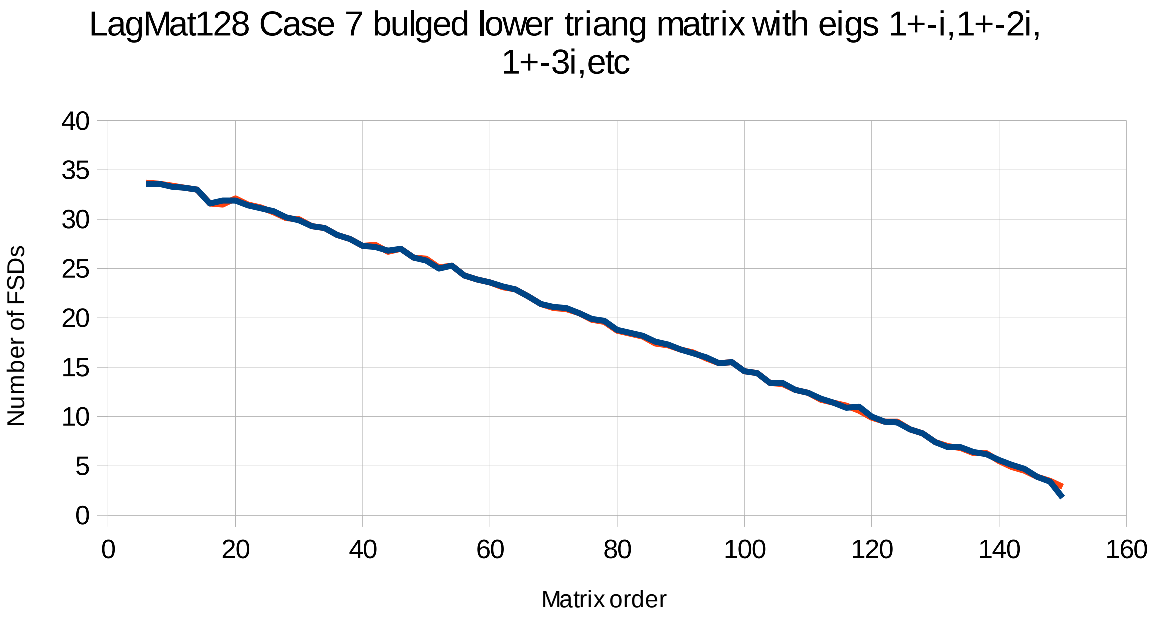
LagMat128 Case 7 bulged lower triang matrix with eigs 1+-i,1+-2i, 1+-3i,etc
Number of FSDs
Matrix order
40
35
30
25
20
15
10
5
0
0
20
40
60
80
100
120
140
160

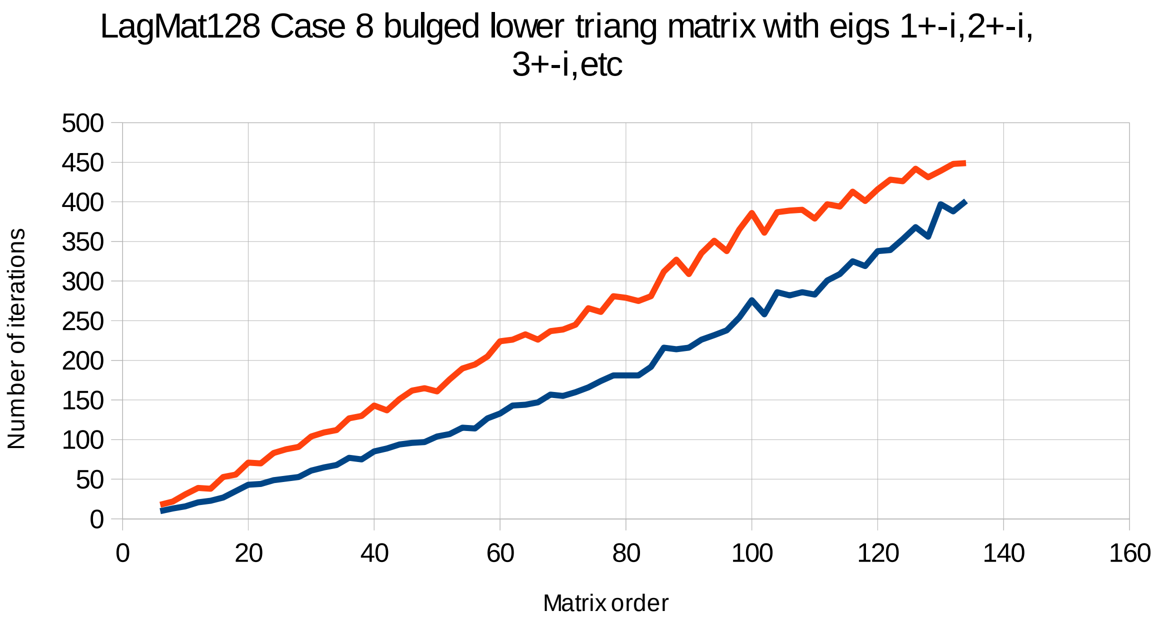
LagMat128 Case 8 bulged lower triang matrix with eigs 1+-i,2+-i, 3+-i,etc
Number of iterations
Matrix order
0
50
100
150
200
250
300
350
400
450
500
0
20
40
60
80
100
120
140
160

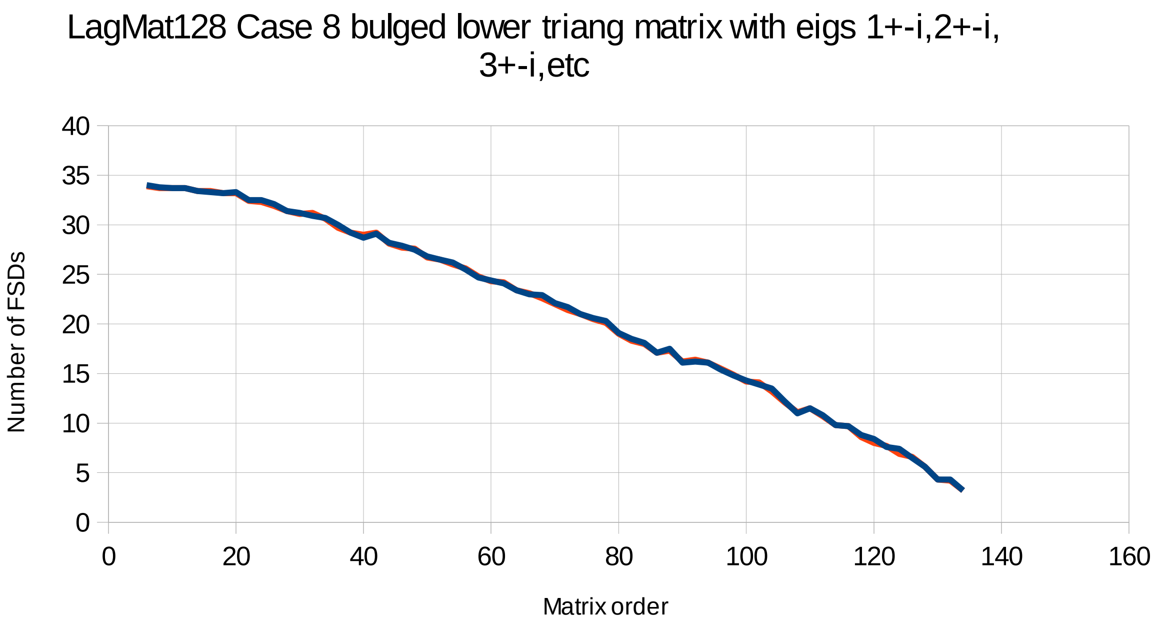
LagMat128 Case 8 bulged lower triang matrix with eigs 1+-i,2+-i, 3+-i,etc
Number of FSDs
Matrix order
0
5
10
15
20
25
30
35
40
0
20
40
60
80
100
120
140
160

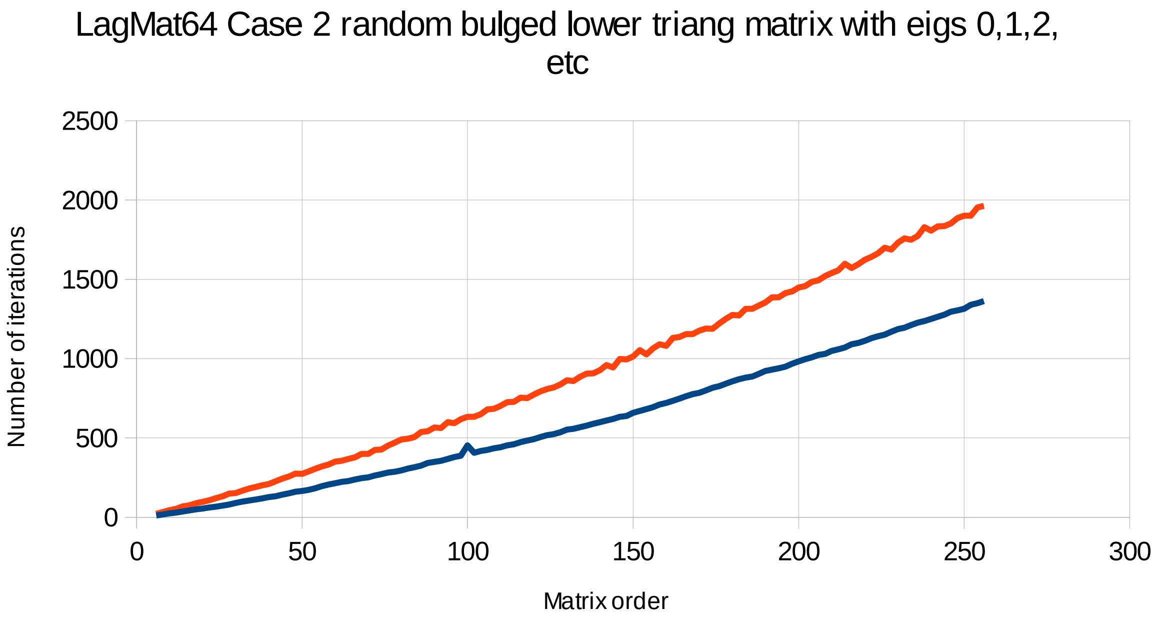
LagMat64 Case 2 random bulged lower triang matrix with eigs 0,1,2, etc
Number of iterations
Matrix order
0
500
1000
1500
2000
2500
0
50
100
150
200
250
300

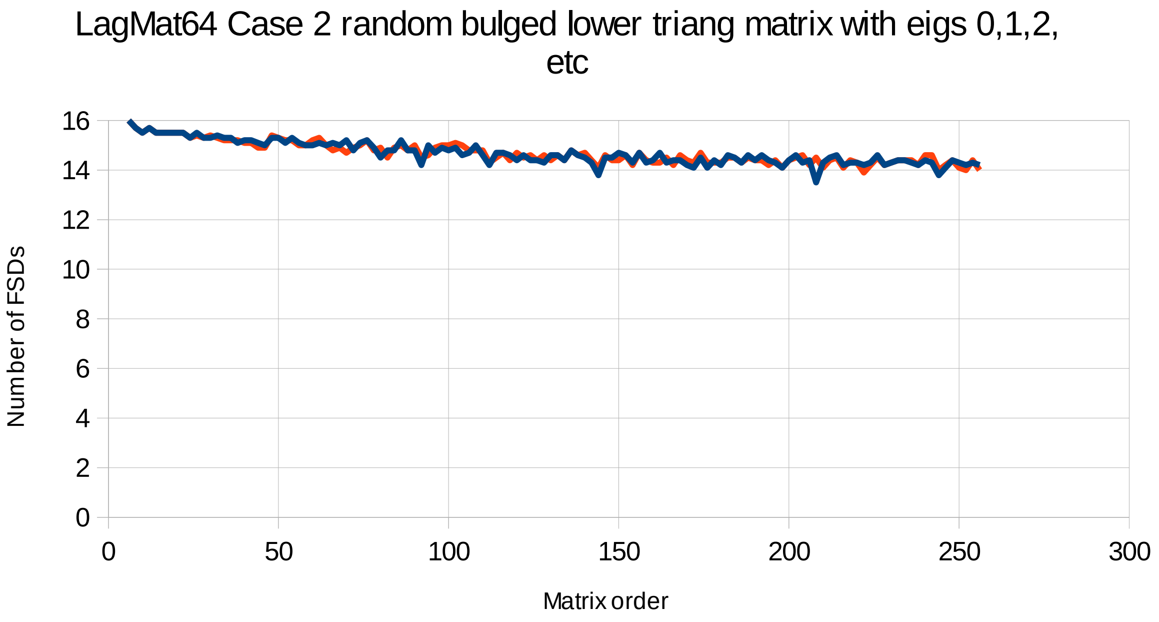
LagMat64 Case 2 random bulged lower triang matrix with eigs 0,1,2, etc
Number of FSDs
16
14
12
10
8
6
4
2
0
0
50
100
150
200
250
300
Matrix order

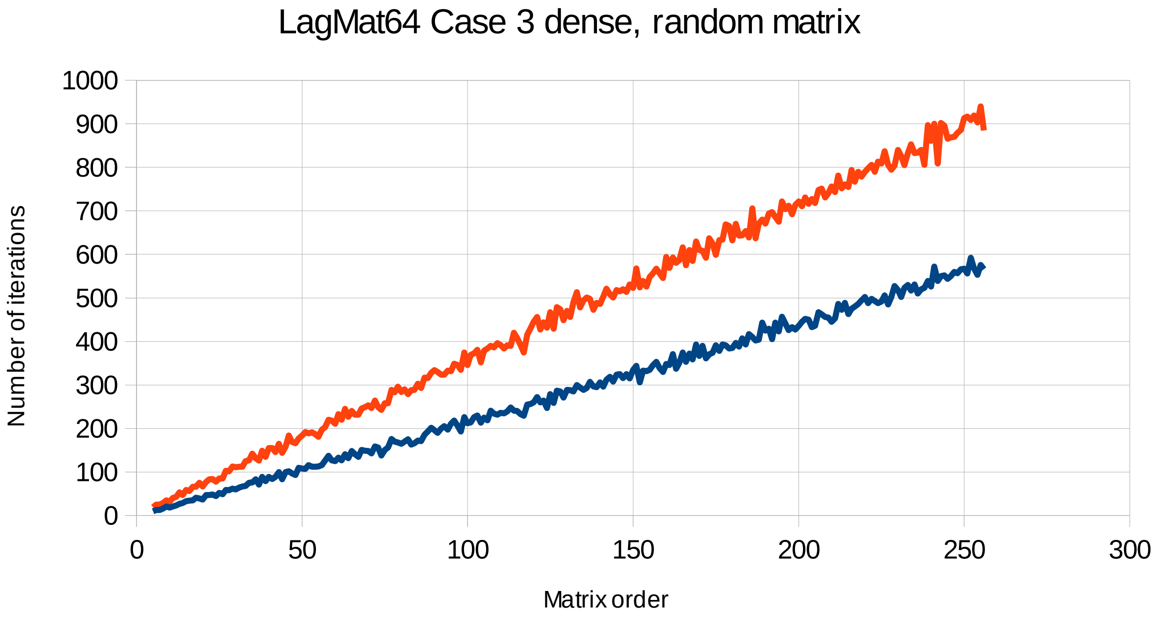
LagMat64 Case 3 dense, random matrix
Number of iterations
1000
900
800
700
600
500
400
300
200
100
0
0
50
100
150
200
250
300
Matrix order

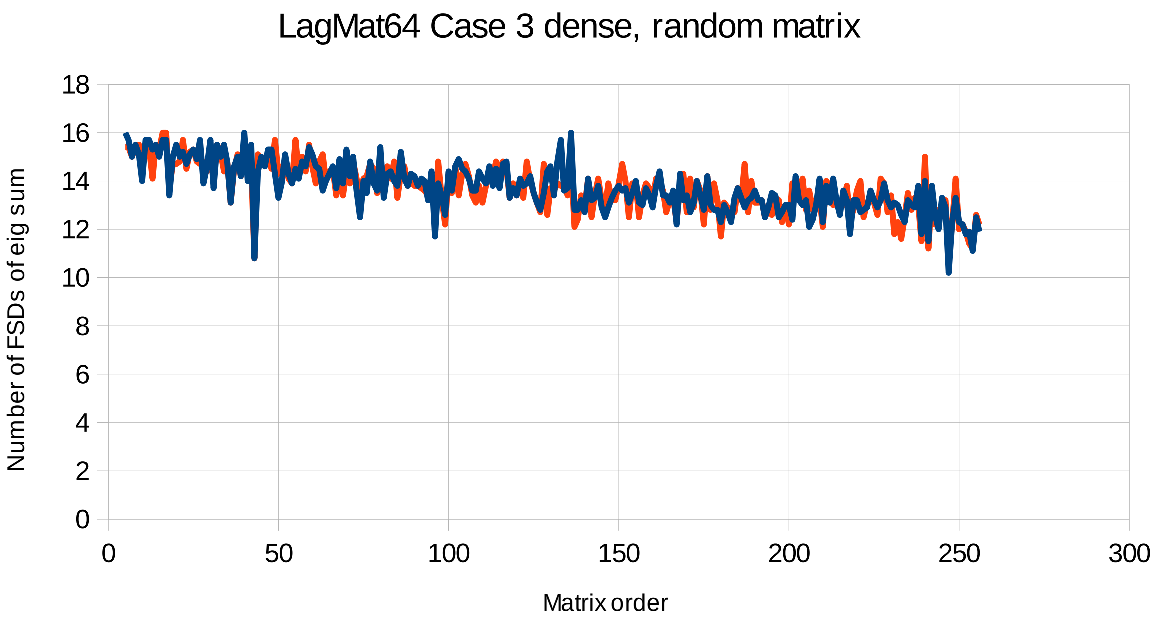
LagMat64 Case 3 dense, random matrix
Number of FSDs of eig sum
Matrix order
0
2
4
6
8
10
12
14
16
18
0
50
100
150
200
250
300

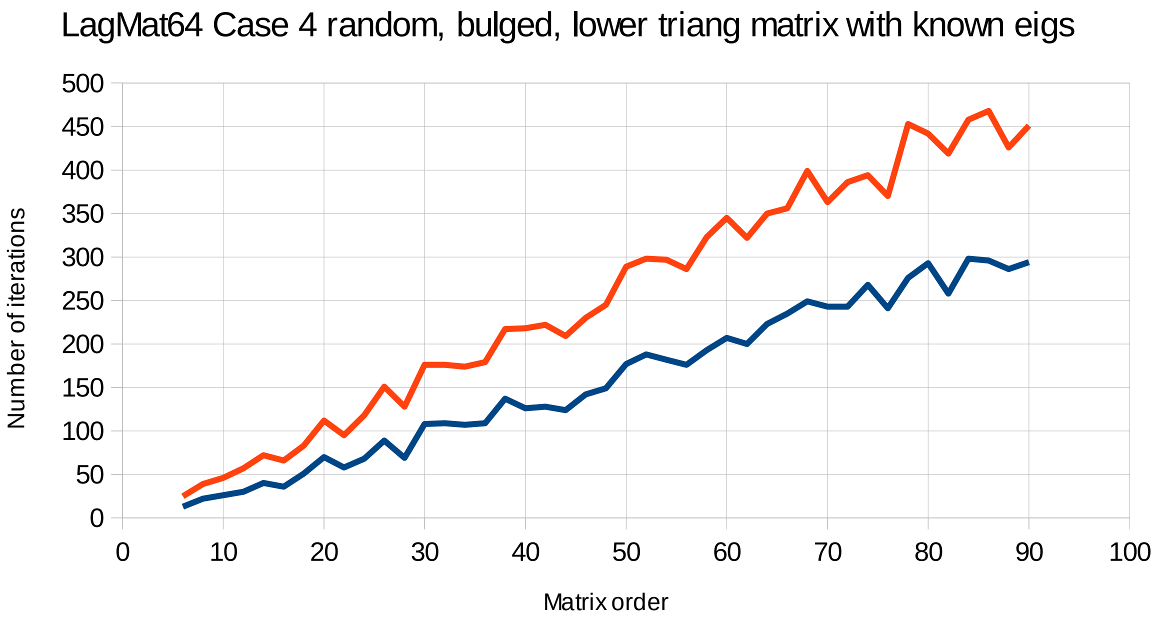
LagMat64 Case 4 random, bulged, lower triang matrix with known eigs
Number of iterations
Matrix order
0
50
100
150
200
250
300
350
400
450
500
0
10
20
30
40
50
60
70
80
90
100

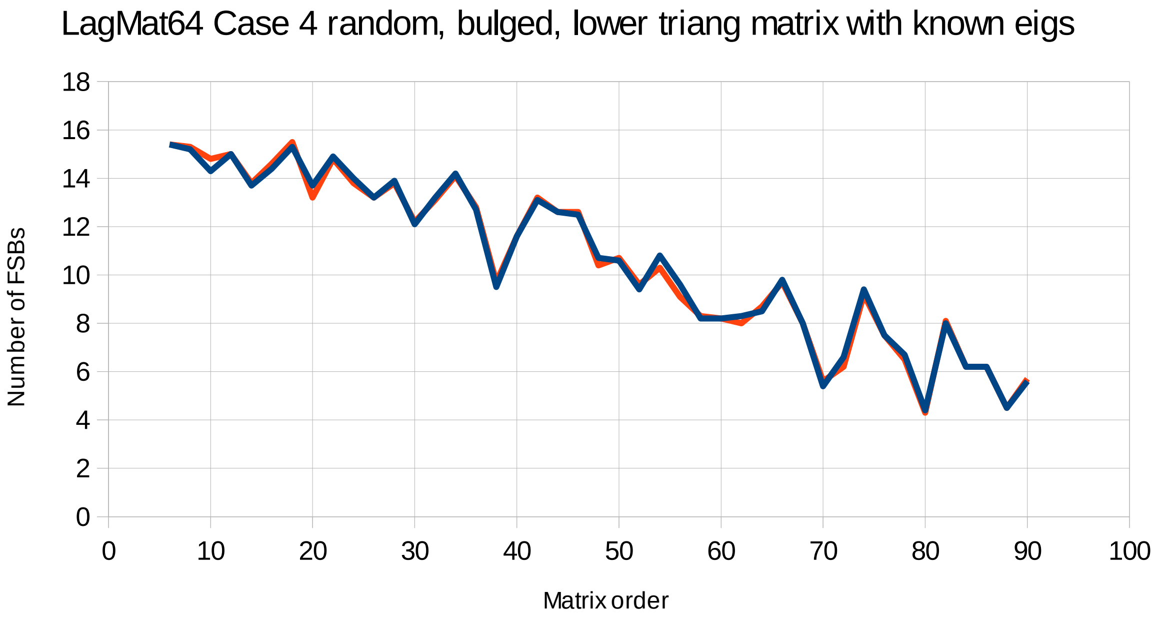
LagMat64 Case 4 random, bulged, lower triang matrix with known eigs
Number of FSBs
Matrix order
0
2
4
6
8
10
12
14
16
18
0
10
20
30
40
50
60
70
80
90
100

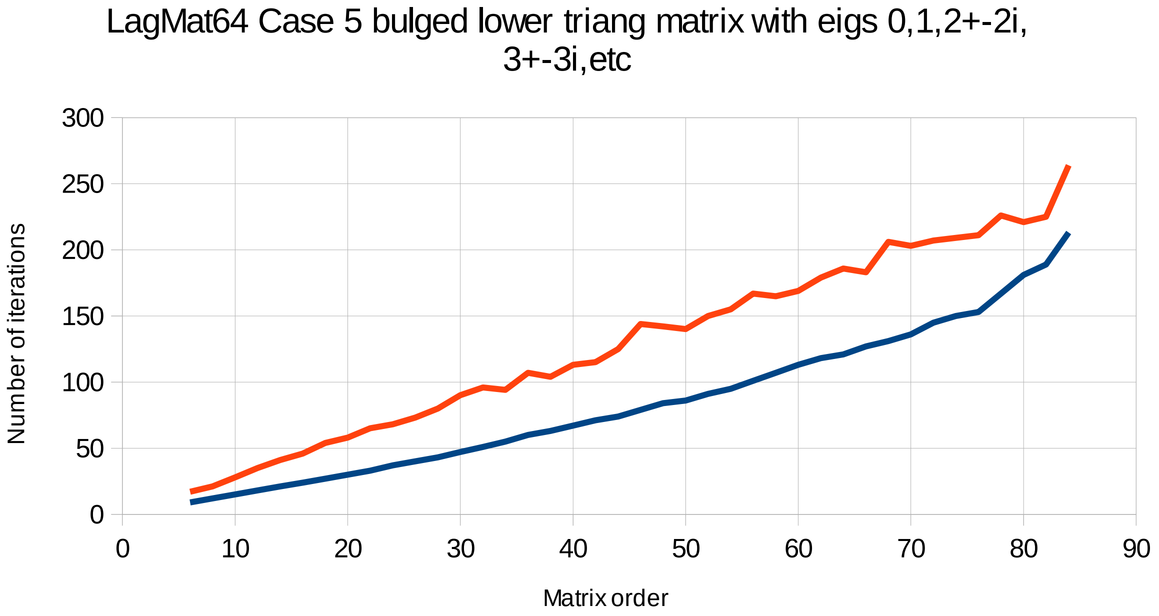

LagMat64 Case 5 bulged lower triang matrix with eigs 0,1,2+-2i, 3+-3i,etc
Number of iterations
Matrix order
0
50
100
150
200
250
300
0
10
20
30
40
50
60
70
80
90

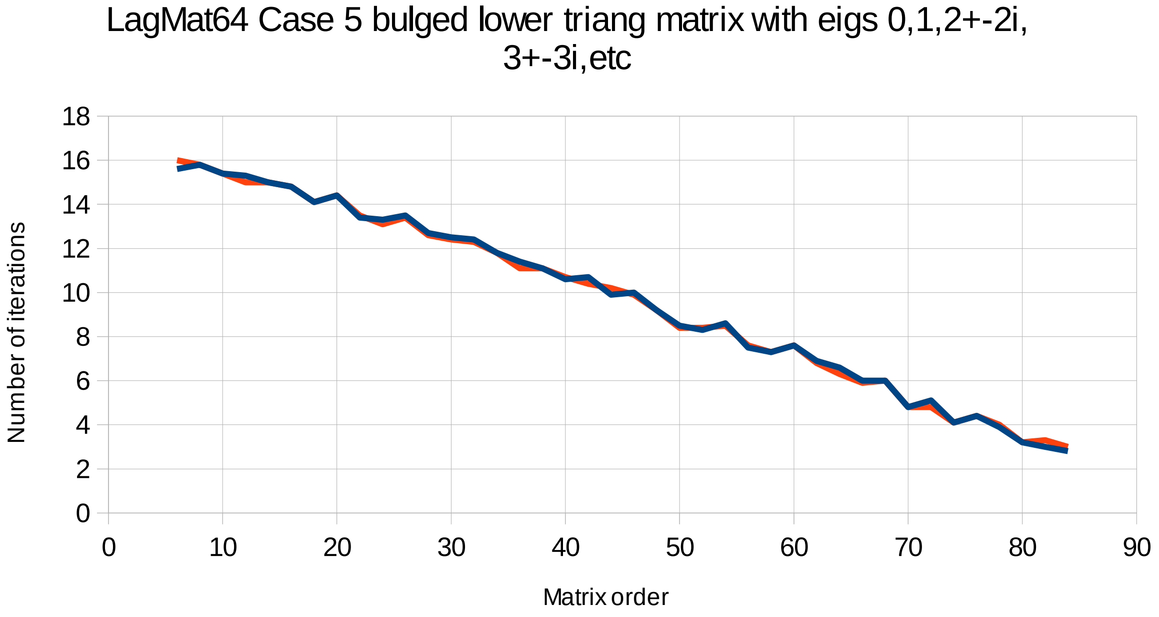

LagMat64 Case 5 bulged lower triang matrix with eigs 0,1,2+-2i, 3+-3i,etc
Number of iterations
Matrix order
0
2
4
6
8
10
12
14
16
18
0
10
20
30
40
50
60
70
80
90

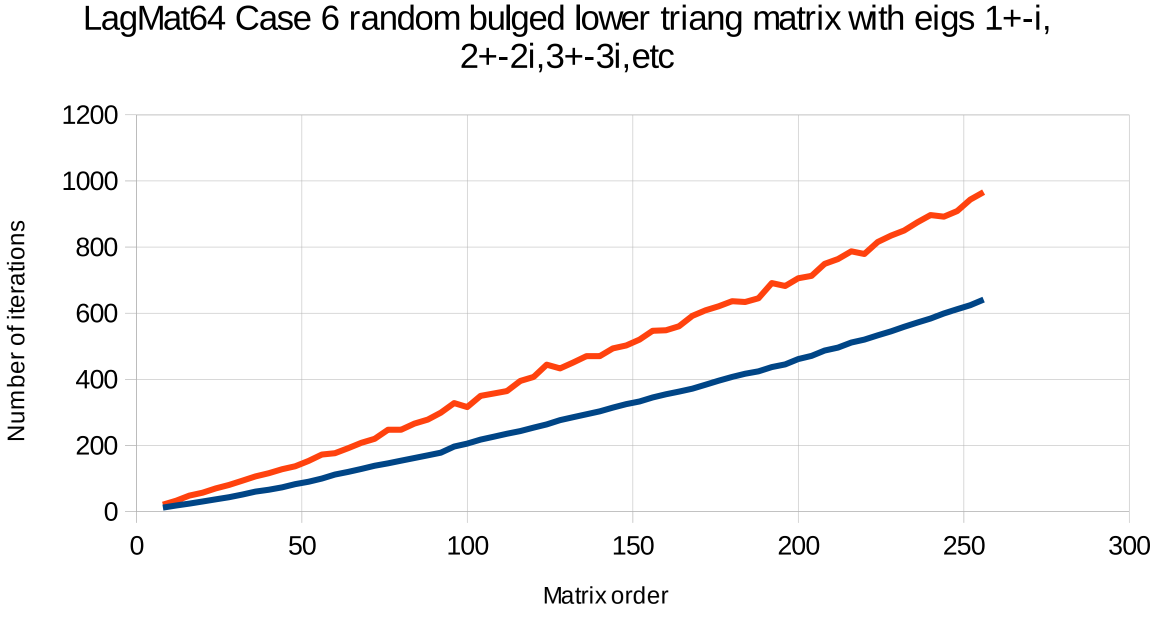

LagMat64 Case 6 random bulged lower triang matrix with eigs 1+-i, 2+-2i,3+-3i,etc
Number of iterations
Matrix order
0
200
400
600
800
1000
1200
0
50
100
150
200
250
300

LagMat64 Case 6 random bulged lower triang matrix with eigs 1+-i, 2+-2i,3+-3i,etc

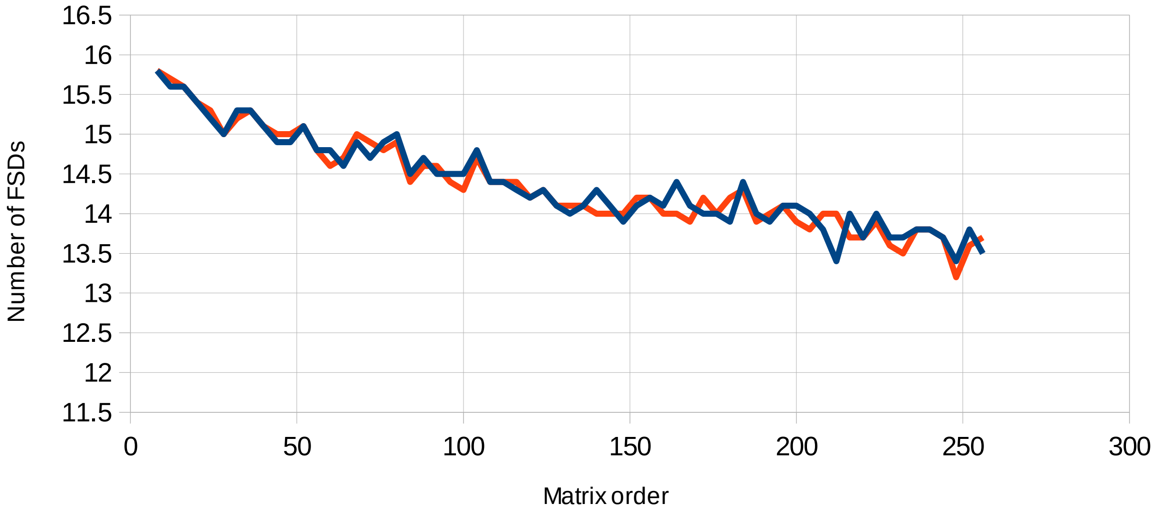

LagMat64 Case 7 bulged lower triang matrix with eigs 1+-i,1+-2i, 1+-3i,etc

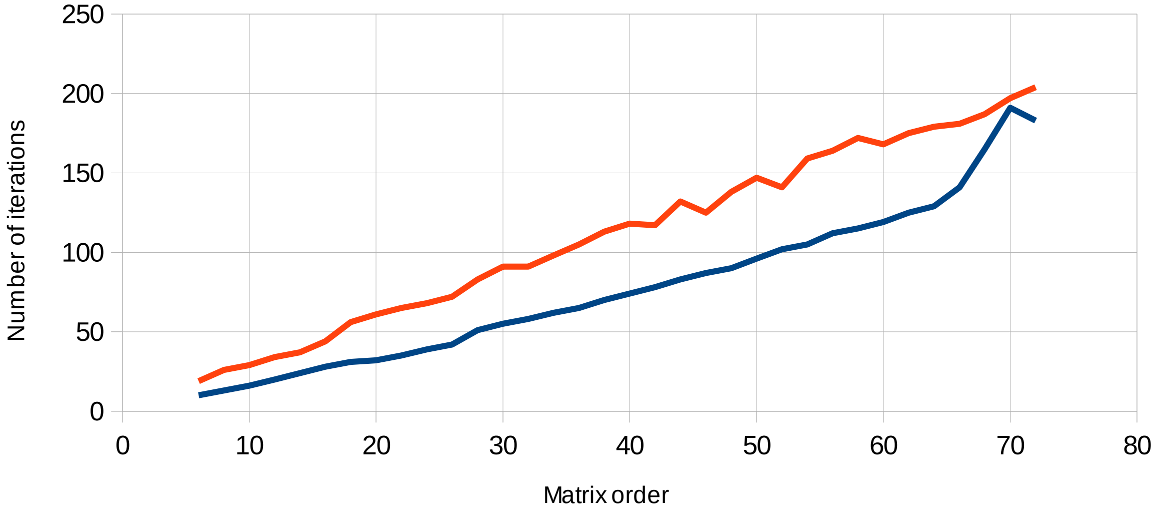

LagMat64 Case 7 bulged lower triang matrix with eigs 1+-i,1+-2i, 1+-3i,etc

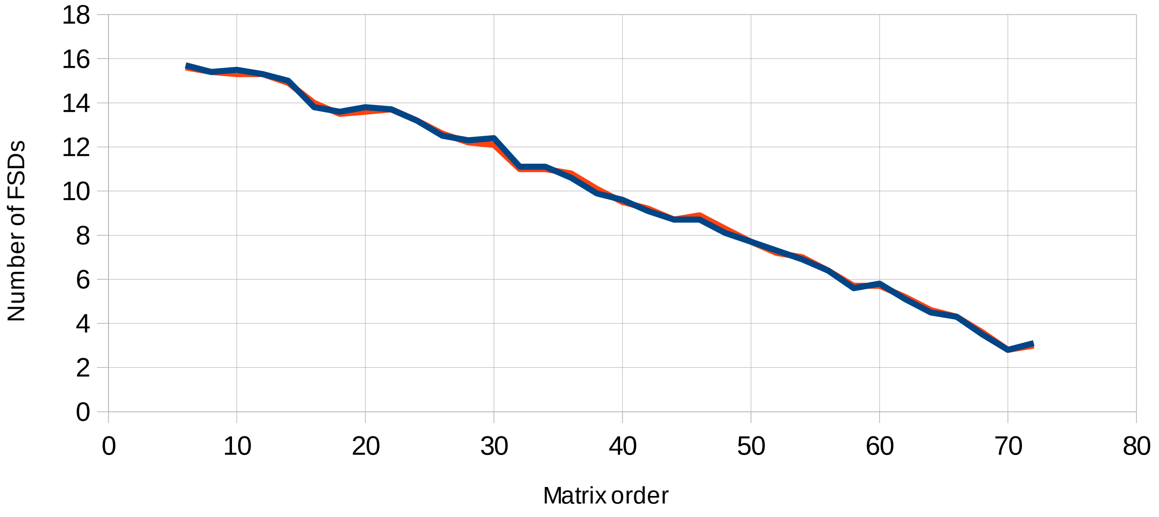

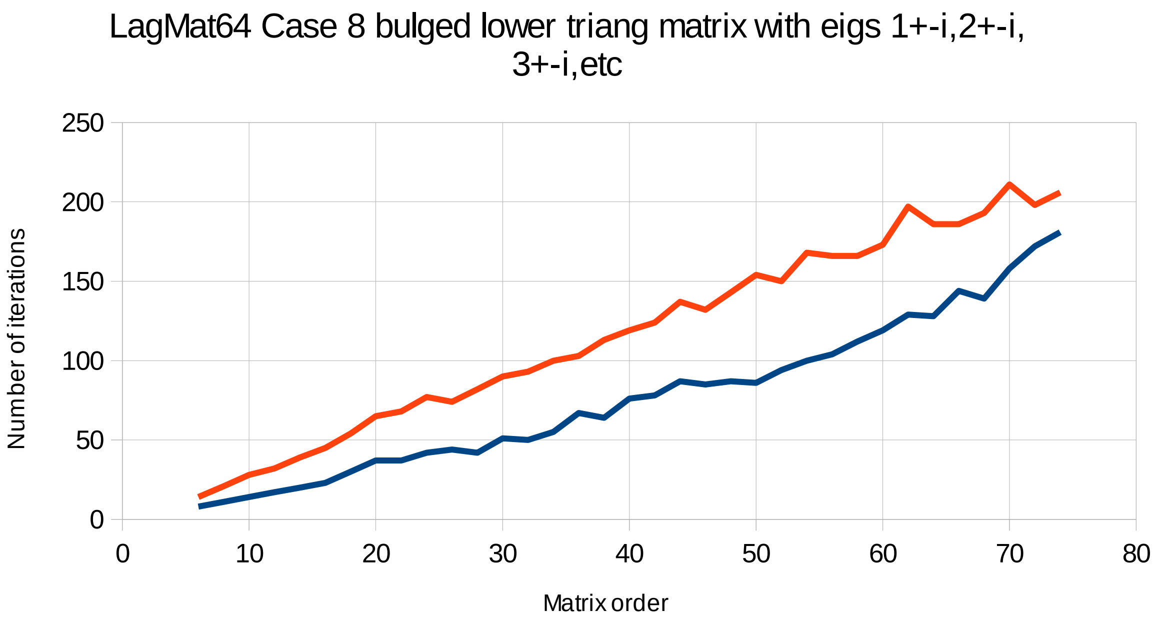
LagMat64 Case 8 bulged lower triang matrix with eigs 1+-i,2+-i, 3+-i,etc
Number of iterations
Matrix order
0
50
100
150
200
250
0
10
20
30
40
50
60
70
80

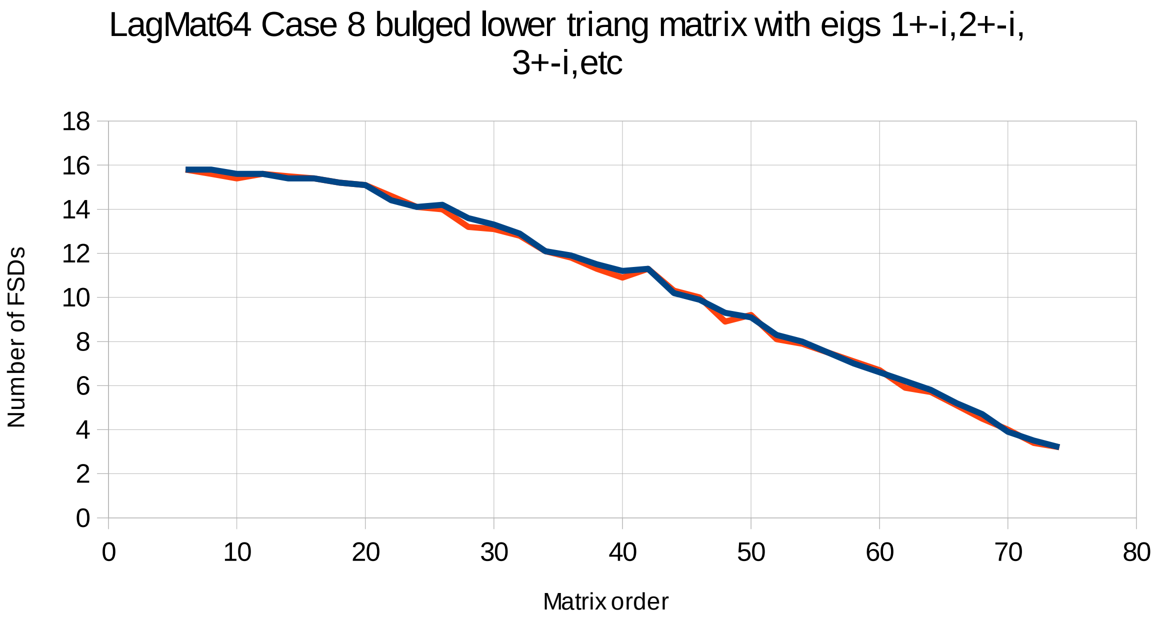
LagMat64 Case 8 bulged lower triang matrix with eigs 1+-i,2+-i, 3+-i,etc
Number of FSDs
Matrix order
0
2
4
6
8
10
12
14
16
18
0
10
20
30
40
50
60
70
80

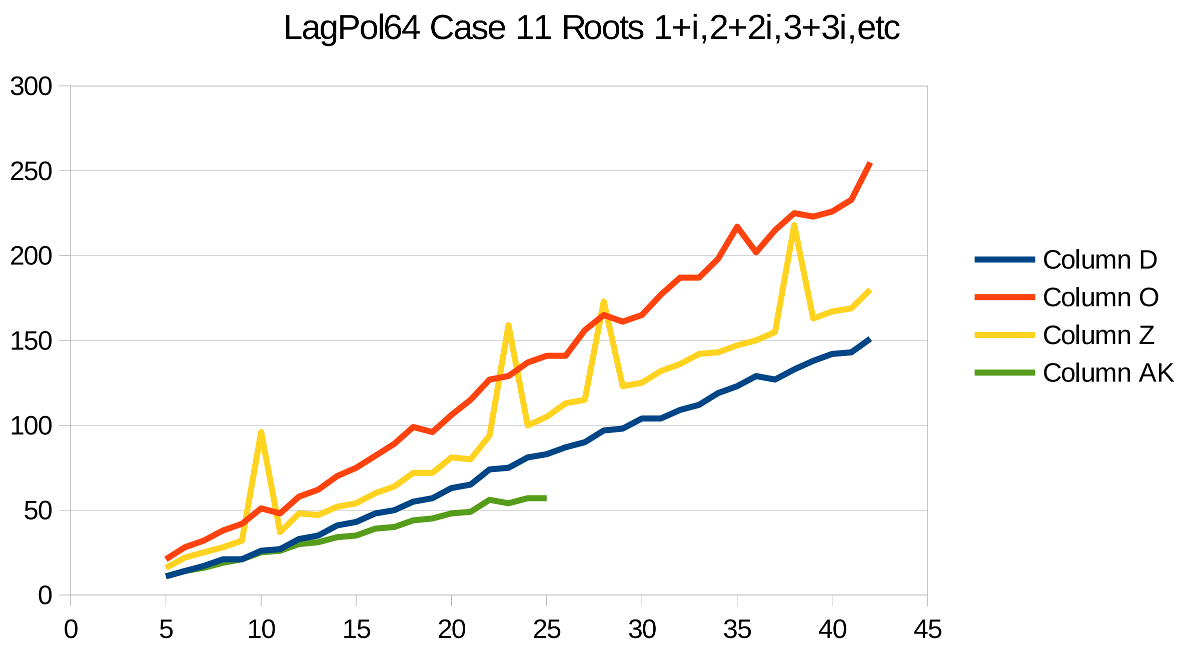
LagPol64 Case 11 Roots 1+i,2+2i,3+3i,etc
0
50
100
150
200
250
300
0
5
10
15
20
25
30
35
40
45
Column D
Column O
Column Z
Column AK

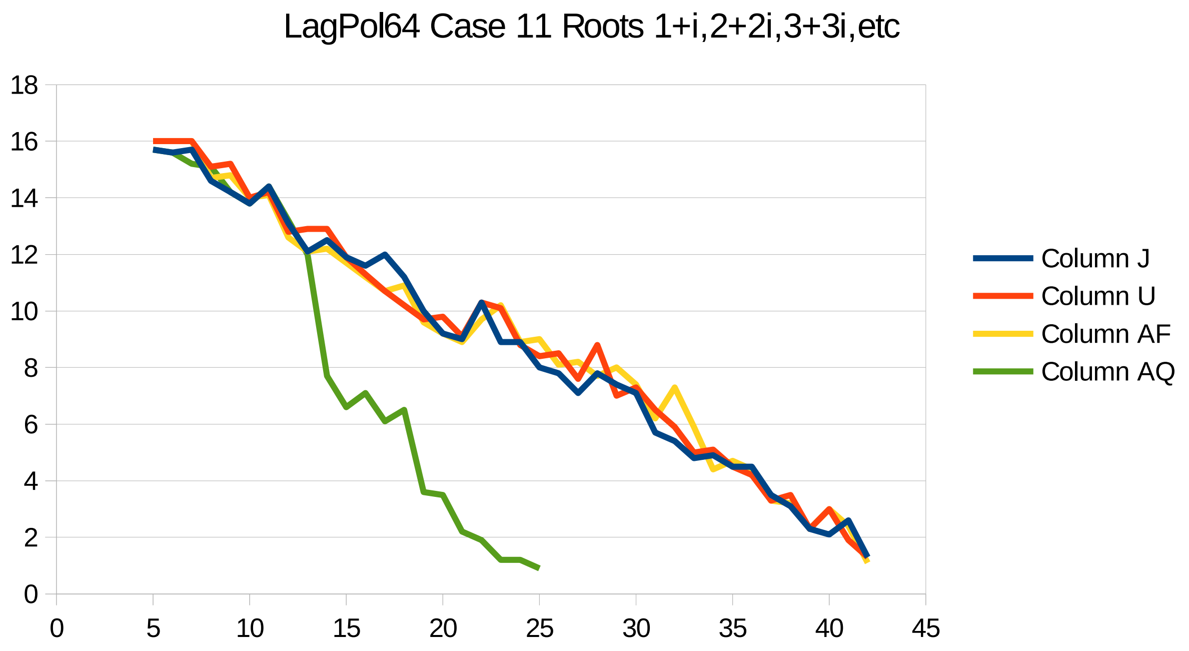
LagPol64 Case 11 Roots 1+i,2+2i,3+3i,etc
0
2
4
6
8
10
12
14
16
18
0
5
10
15
20
25
30
35
40
45
Column J
Column U
Column AF
Column AQ

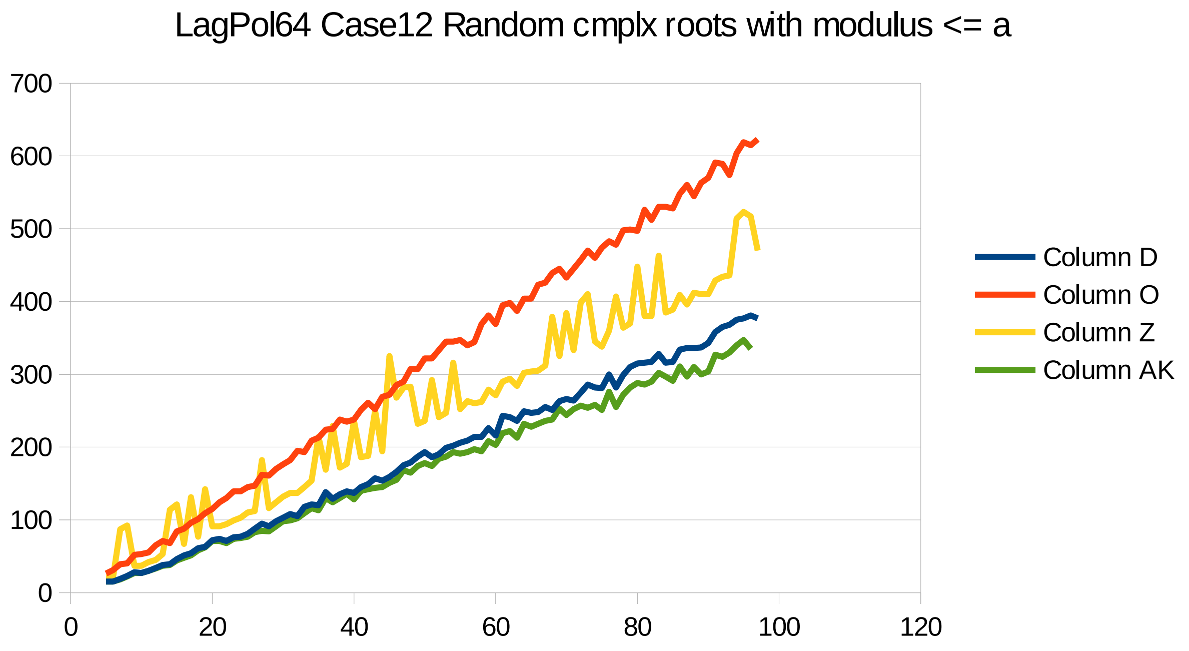
LagPol64 Case12 Random cmplx roots with modulus <= a
0
100
200
300
400
500
600
700
0
20
40
60
80
100
120
Column D
Column O
Column Z
Column AK

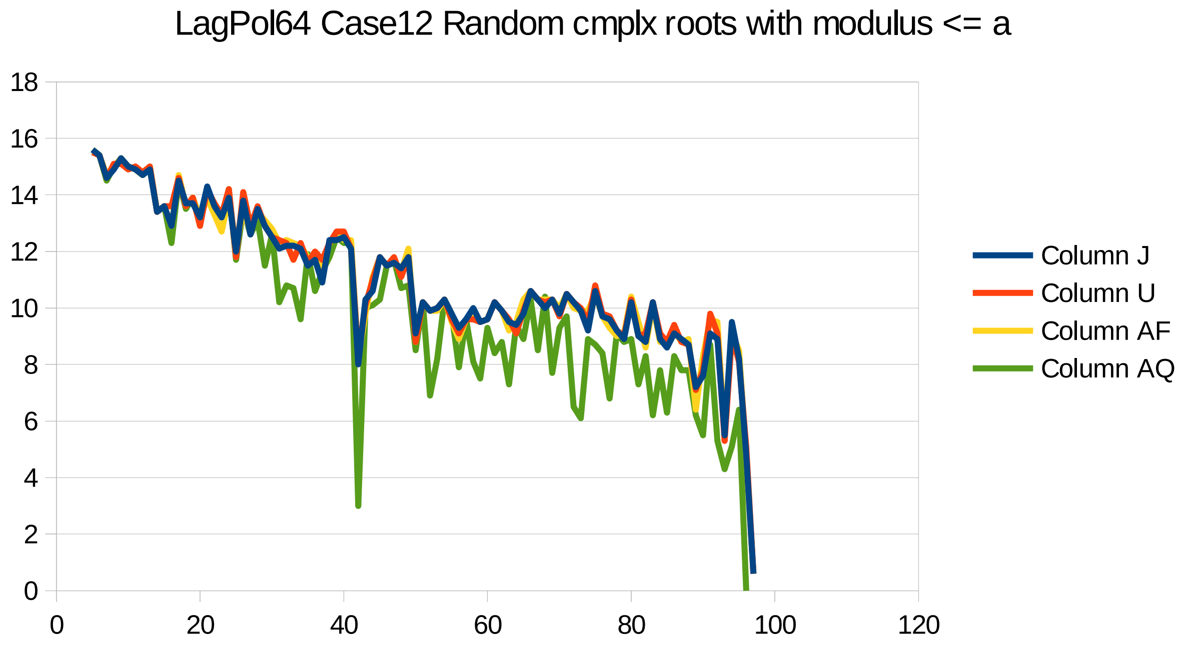
LagPol64 Case12 Random cmplx roots with modulus <= a
0
2
4
6
8
10
12
14
16
18
0
20
40
60
80
100
120
Column J
Column U
Column AF
Column AQ

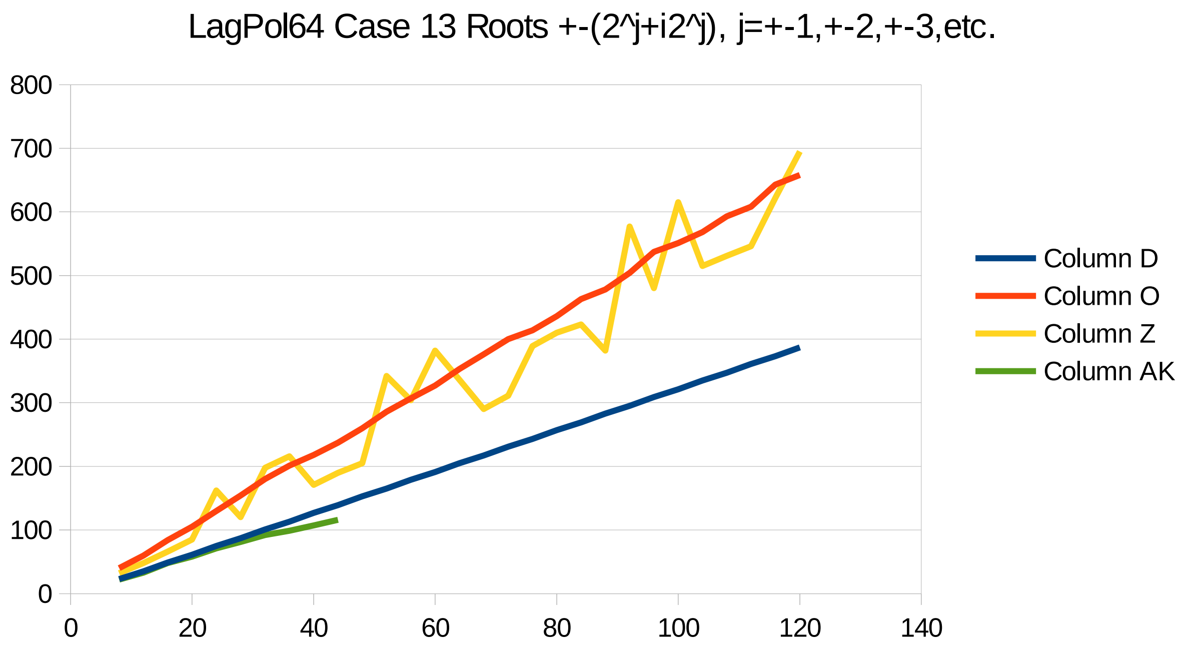
LagPol64 Case 13 Roots +-(2^j+i2^j), j=+-1,+-2,+-3,etc.
800
700
600
500
400
300
200
100
0
0
20
40
60
80
100
120
140
Column D
Column O
Column Z
Column AK

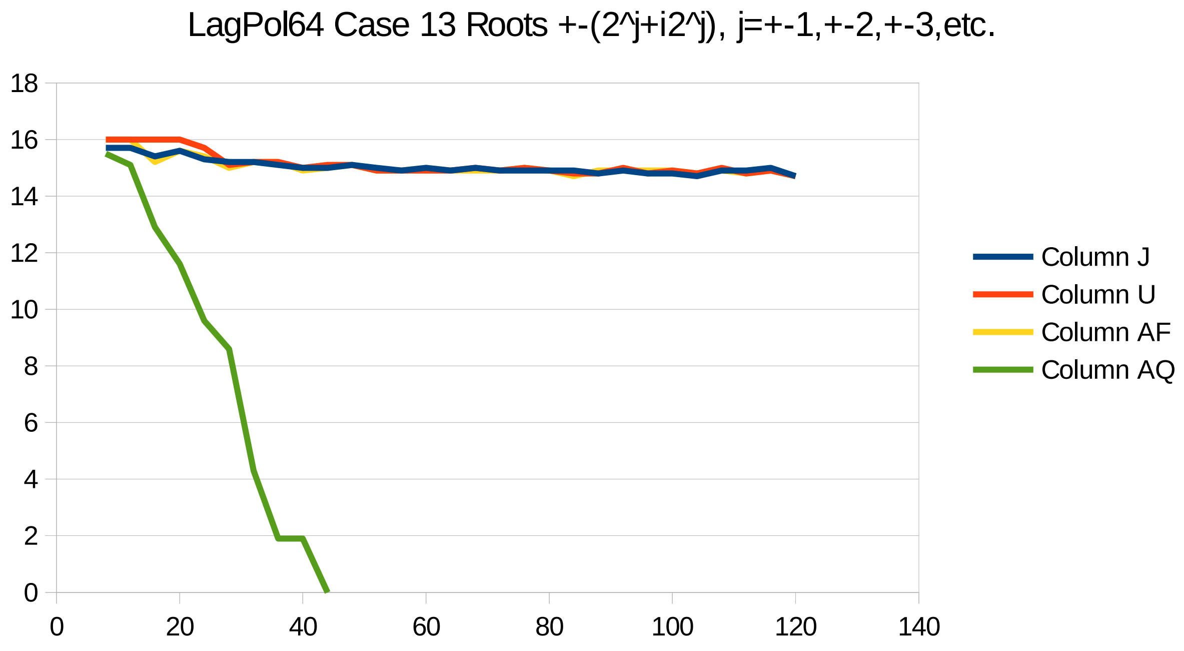
LagPol64 Case 13 Roots +-(2^j+i2^j), j=+-1,+-2,+-3,etc.
18
16
14
12
10
8
6
4
2
0
0
20
40
60
80
100
120
140
Column J
Column U
Column AF
Column AQ

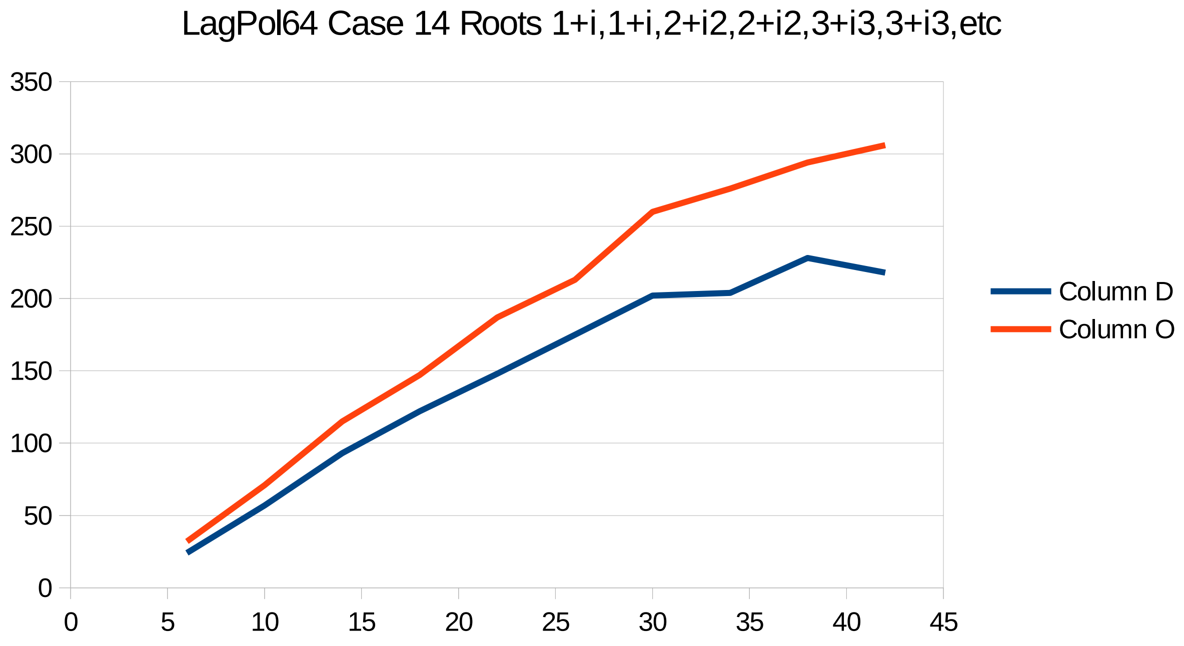
LagPol64 Case 14 Roots 1+i,1+i,2+i2,2+i2,3+i3,3+i3,etc
350
300
250
200
150
100
50
0
0
5
10
15
20
25
30
35
40
45
Column D
Column O

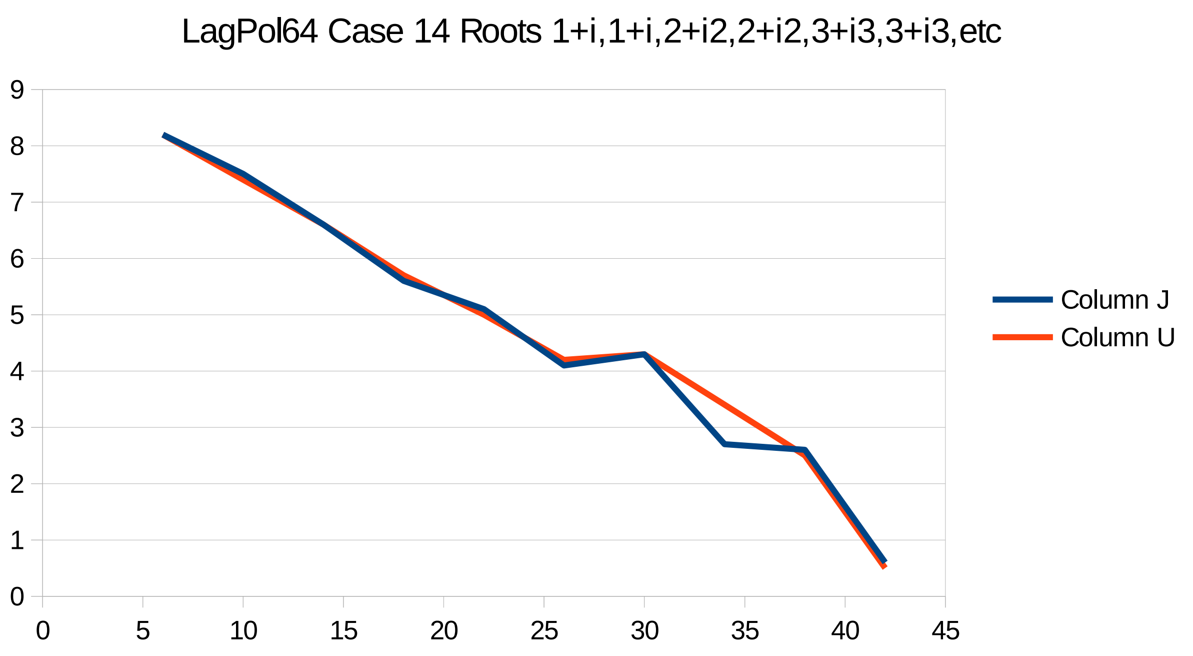
LagPol64 Case 14 Roots 1+i,1+i,2+i2,2+i2,3+i3,3+i3,etc
0
1
2
3
4
5
6
7
8
9
0
5
10
15
20
25
30
35
40
45
Column J
Column U

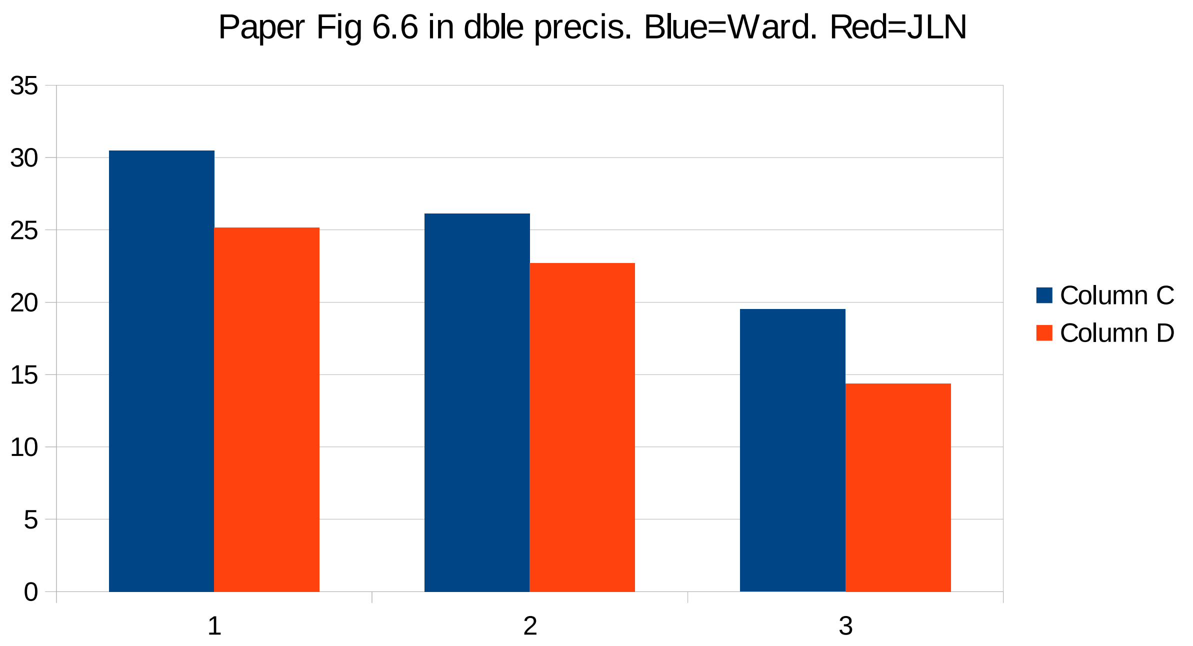
Paper Fig 6.6 in dble precis. Blue=Ward. Red=JLN
0
5
10
15
20
25
30
35
1
2
3
Column C
Column D
